# Age-related long-term response in rat thyroid tissue and plasma after internal low dose exposure to ^131^I

**DOI:** 10.1038/s41598-022-06071-4

**Published:** 2022-02-08

**Authors:** Malin Larsson, Nils-Petter Rudqvist, Johan Spetz, Toshima Z. Parris, Britta Langen, Khalil Helou, Eva Forssell-Aronsson

**Affiliations:** 1 Department of Medical Radiation Sciences, Institute of Clinical Sciences, Sahlgrenska Center for Cancer Research, Sahlgrenska Academy, University of Gothenburg, 413 45 Gothenburg, Sweden; 2 Department of Thoracic/Head and Neck Medical Oncology, University of Texas MD Anderson, Houston, TX 77030 USA; 3 Department of Immunology, University of Texas MD Anderson, Houston, TX 77030 USA; 4 John B. Little Center for Radiation Sciences, Harvard T. H. Chan School of Public Health, Boston, MA 02115 USA; 5 Department of Oncology, Institute of Clinical Sciences, Sahlgrenska Center for Cancer Research, Sahlgrenska Academy, University of Gothenburg, 413 45 Gothenburg, Sweden; 6 UT Department of Radiation Oncology, Division of Molecular Radiation Biology, UT Southwestern Medical Center, 2201 Inwood Rd., Dallas, TX 75390 USA; 7 Department of Medical Physics and Biomedical Engineering, Sahlgrenska University Hospital, 413 45 Gothenburg, Sweden

**Keywords:** Biological techniques, Cancer, Cell biology, Biomarkers, Physics

## Abstract

^131^I is used clinically for therapy, and may be released during nuclear accidents. After the Chernobyl accident papillary thyroid carcinoma incidence increased in children, but not adults. The aims of this study were to compare ^131^I irradiation-dependent differences in RNA and protein expression in the thyroid and plasma of young and adult rats, and identify potential age-dependent biomarkers for ^131^I exposure. Twelve young (5 weeks) and twelve adult Sprague Dawley rats (17 weeks) were i.v. injected with 50 kBq ^131^I (absorbed dose to thyroid = 0.1 Gy), and sixteen unexposed age-matched rats were used as controls. The rats were killed 3–9 months after administration. Microarray analysis was performed using RNA from thyroid samples, while LC–MS/MS analysis was performed on proteins extracted from thyroid tissue and plasma. Canonical pathways, biological functions and upstream regulators were analysed for the identified transcripts and proteins. Distinct age-dependent differences in gene and protein expression were observed. Novel biomarkers for thyroid ^131^I exposure were identified: (PTH), age-dependent dose response (CA1, FTL1, PVALB (youngsters) and HSPB6 (adults)), thyroid function (*Vegfb* (adults)). Further validation using clinical samples are needed to explore the role of the identified biomarkers.

## Introduction

Thyroid diseases are routinely examined or treated with ^131^I, due to natural physiological uptake of iodine. However, ionizing radiation can also induce cancer in normal cells. During the Chernobyl accident, large amounts of radioactive nuclides were released into the atmosphere, including over 1.8 EBq of ^131^I activity^1^. The absorbed dose to thyroid from ^131^I was then higher in children in the most contaminated areas (ca 900 mGy and 170 mGy for evacuated and non-evacuated children and adolescents) than adults, which may partly explain why more papillary thyroid cancer (PTC) cases (> 5000 during 1992–2005) were detected in children but not adults^2^. Children are also likely more sensitive to radiation than adults, due to higher more active cellular proliferation, with cells more often in sensitive cell cycle phases (M and G2 phase)^3,4^. Epidemiological studies on thyroid cancer risks have been performed and reviewed^5,6^. Furthermore, children and young adults that received ^131^I for diagnostic purposes had a higher risk of developing secondary malignancies, besides having longer expected lifespans^7^. However, a large Swedish study demonstrated no excess risk for thyroid cancer in adults after diagnostic exposure to ^131^I^8^*.* Nevertheless, our understanding of the long-term effects of ^131^I exposure in thyroid tissue is limited and more knowledge is needed.

In recent years, efforts were made to identify tissue-specific molecular markers (biomarkers), e.g. proteins and RNA transcripts that can predict previous radiation exposure, altered thyroid function, and cancer induction. Traditionally, immunohistopathology was the method of choice^9^. Lately, several alternative methods were introduced, e.g. genetic profiles, including single nucleotide polymorphisms (SNPs), and gene and protein expression^10–14^. To study the impact of irradiation on thyroid tissue, tumour material from patients irradiated after the Chernobyl accident were analysed. Genotyping of PTC tissue showed that SNPs in *ATM* exon 39 and *XRCC1* exon 10 were potential biomarkers of decreased PTC risk in adults, and *ATM* IVS22-77 and *TP53* codon 72 were associated with radiation exposure^15^. Based on PTC tissue from children, the CLIP2 protein was proposed as a radiation-induced biomarker^12,16^. The thyroglobulin (TG) protein level in blood was previously proposed as a biomarker for externally irradiated thyroid during childhood^17,18^. TG blood levels may also be related to thyroid size and function rather than type of thyroid malignancy, and its potential as a cancer biomarker needs to be further evaluated^19^.

We have previously proposed the *Agpat9, Klk1, Klk1b* family, *Plau, Prf1,* and *S100a8* genes as biomarkers for ^131^I exposure in adult mouse thyroid tissue using transcriptomic and proteomic analyses^20–23^. We also showed that circadian rhythm affected gene expression patterns in the thyroid, with a strong association with the kallikrein gene family^21^. Recently, we investigated the biological effects on thyroid tissue nine months after ^131^I injection in young rats (thyroid absorbed dose of 0.01–1 Gy)^24^. Several significant ^131^I exposure-related RNA transcripts (*Afp* and *RT1-Bb*) and proteins (ARF3, DLD, IKBKB, NONO, RAB6A, RPN2, and SLC25A5), and absorbed dose-related biomarkers (APRT, DSG4, LDHA, and TGM3) were identified. Candidate biomarkers for changes in thyroid function were also proposed: ACADL, SORBS, TPO and TG proteins. These data demonstrate the importance of directly comparing age-related long-term differences in radiobiological response to ^131^I exposure.

The aims of this work were to (a) identify differences in expression patterns on the RNA and protein levels in young and adult rats exposed to ^131^I, and (b) identify potential biomarkers related to ^131^I exposure and thyroid function.

## Results

The six groups of Sprague Dawley rats exposed to ^131^I are hereafter denoted as 3 months young (Y3), 6 months young (Y6), 9 months young (Y9), 3 months adult (A3), 6 months adult (A6), and 9 months adult (A9) rats. When combing data from two or three groups, abbreviations were used, such as Y3 + Y6 as a shortening for the Y3 and Y6 groups together.

### Time-related RNA expression profiles in irradiated versus non-irradiated young rats

**Figure 1:**
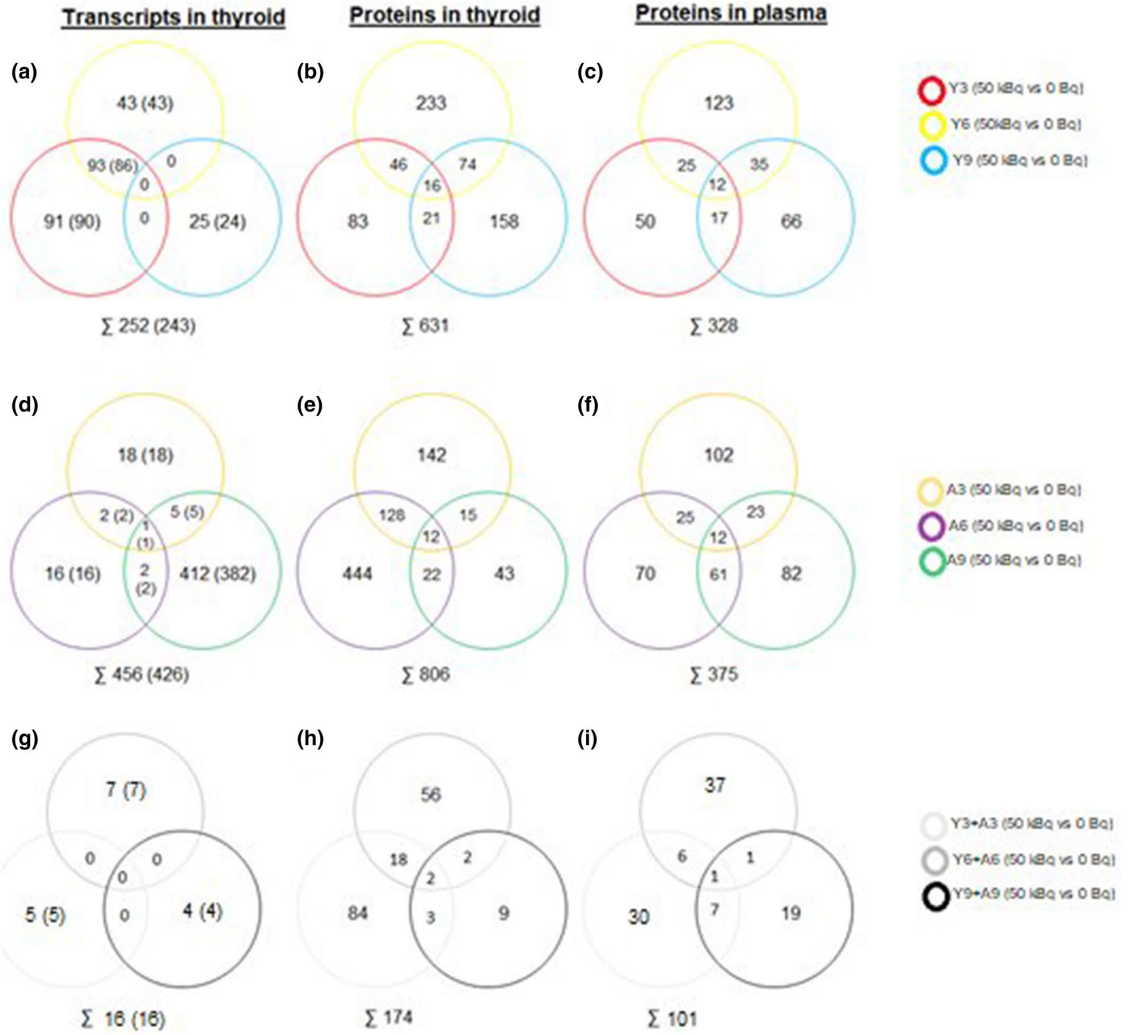
A schematic illustration of the distribution of statistically significant transcripts and proteins in rats exposed to ^131^I. Venn diagrams displaying the number of (**a**, **d**, **g**) transcripts identified in thyroid tissue (**b**, **e**, **h**) proteins identified in thyroid tissue and (**c**, **f**, **i**) proteins identified in plasma. Panels (**a**–**c**) and (**d**–**f**) represent young (Y) rats and adult (A) rats, respectively, while (**g**–**i**) show transcripts or proteins identified at three, six or nine months after irradiation in both young and adult rats. When reporting number on transcripts, the number of related genes id given in parenthesis. In legends, Y denotes young and A adult rats, and the figure gives the number of months after injection of ^131^I.

**Figure 2:**
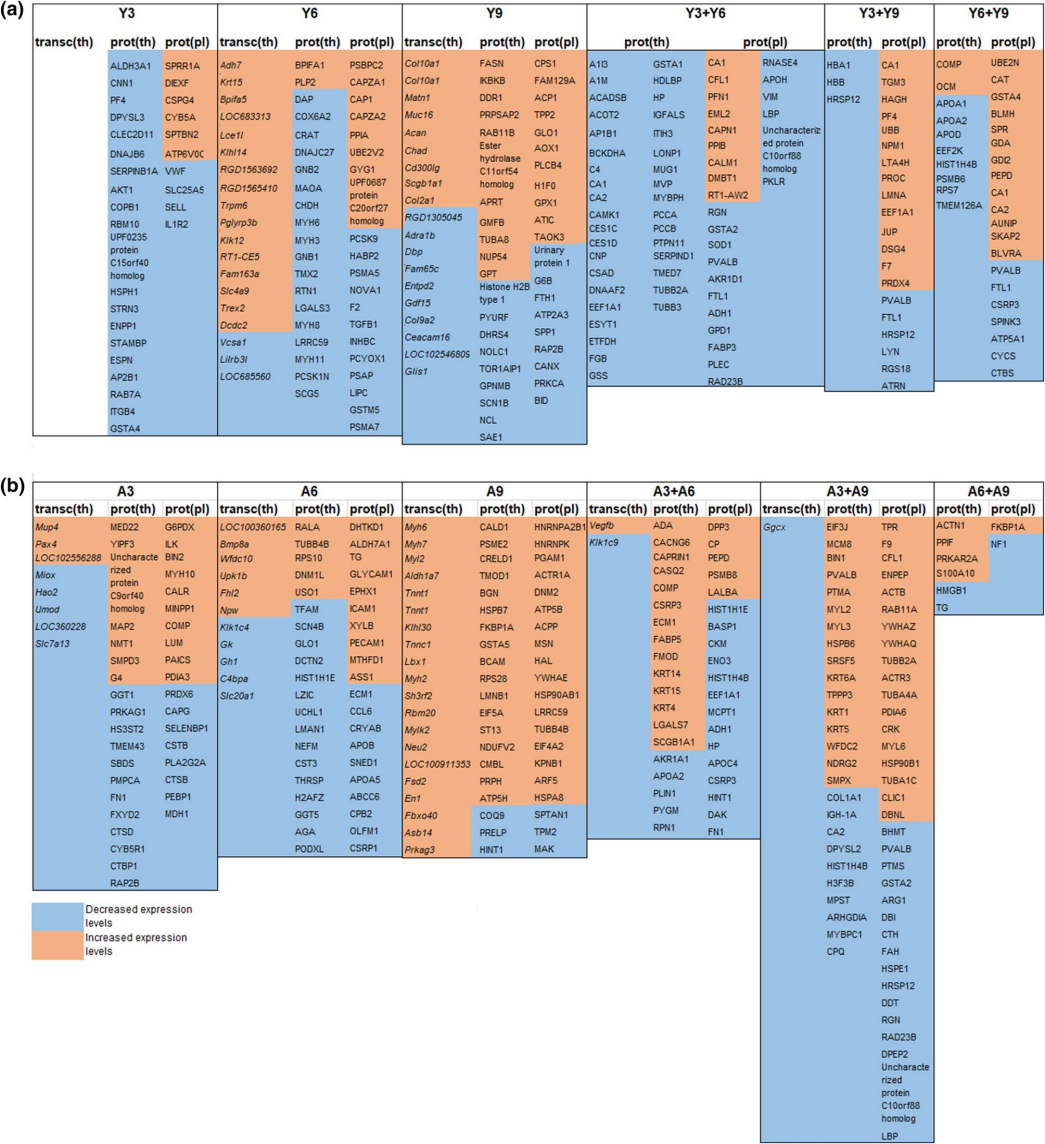
The 20 transcripts and proteins with highest expression levels in thyroid tissue, and plasma for (**a**) young and (**b**) adult rats exposed to ^131^I. Red or blue colour represents increased or decreased expression levels, respectively Y stands for young, A for adult, transc(th) for transcripts in thyroid tissue, prot(th) for proteins in thyroid and prot(pl) for proteins in plasma.

RNA microarray analysis identified 252 differentially regulated transcripts (corresponding to 243 genes) in the irradiated thyroids of young rats (Fig. 1a), of which 91, 43, and 25 transcripts (90, 43, and 24 genes) were unique for the Y3, Y6, and Y9 groups, respectively (Supplementary Table S1). Twenty RNA transcripts with the highest differential regulation are presented in Fig. 2a. The *Tpo* (Y3), *Klhl14*and *Tg* (Y6) genes encodes for the TPO, KLHL14 and TG proteins that are involved in thyroid function according to the Human Protein Atlas (HPA) (Supplementary Table S2). No RNA transcript was differentially regulated in all three groups for young individuals. However, 93 RNA transcripts (86 genes) were commonly regulated in the Y3 and Y6 groups (Fig. 1a).

**Figure 3:**
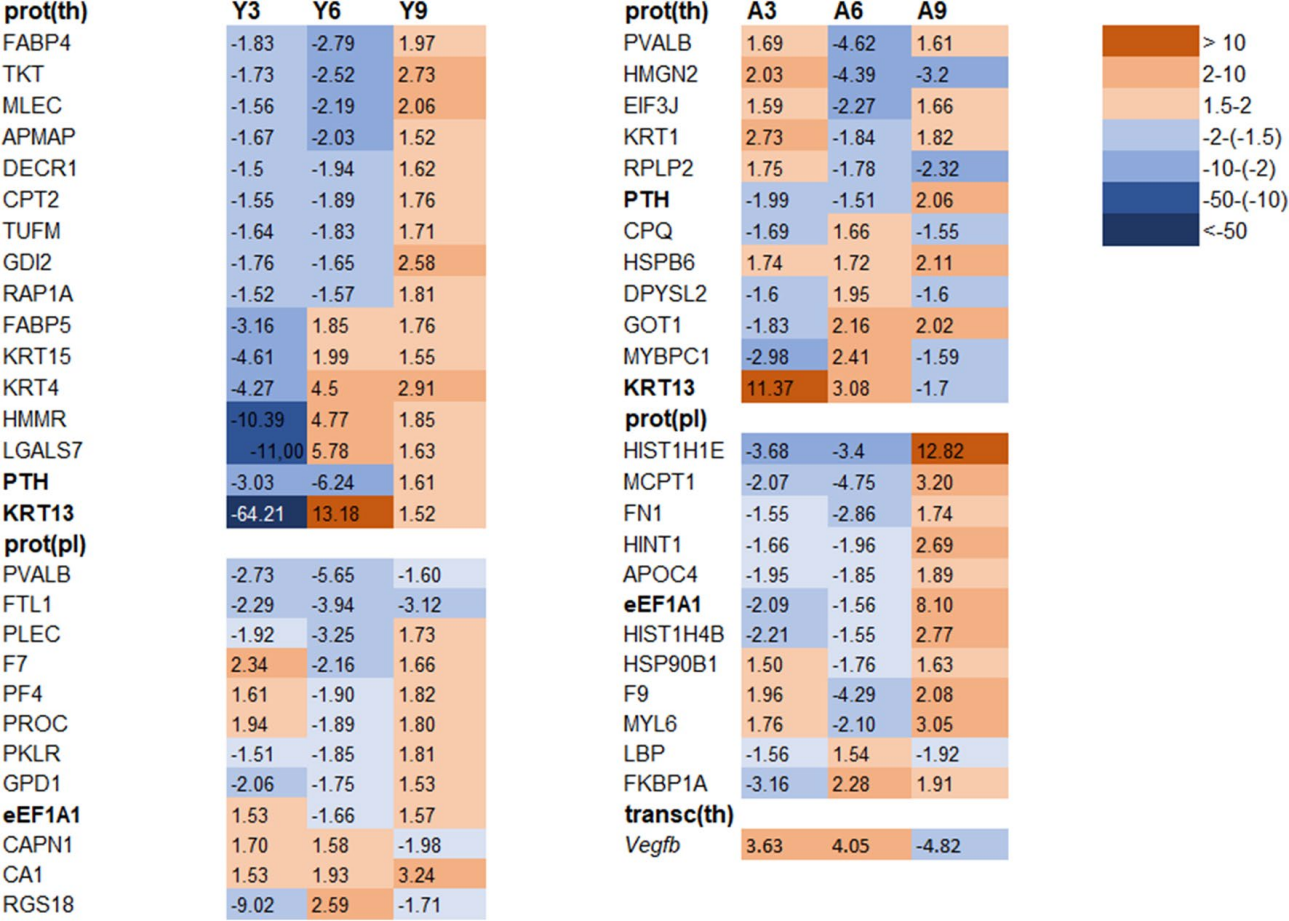
Common transcripts and proteins in young and adult rats at all three time points, respectively. The proteins marked with bold are common for both young and adult individuals. Transcripts in thyroid transc(th), protein in thyroid prot(th) and proteins in plasma prot(pl) are represented and Y = young and A = adult. Red represents increased expression, and blue represents decreased expression. A darker colour represents higher/lower expression.

Mass spectrometry analysis identified 631 proteins associated with ^131^I exposure in young rat thyroid tissue (Fig. 1b), of which 83, 233, and 158 unique proteins were identified in the Y3, Y6, and Y9 groups, respectively (Supplementary Table S3). Only the OCLN (Y6) and SORBS2 (Y9) proteins were highly associated with thyroid function according to HPA (Supplementary Table S2). Twenty proteins with the highest differential expression levels are presented in Fig. 2b. In total, 141 proteins were identified in at least two groups, of which ACADL was thyroid-related (Y6 + Y9) (Supplementary Table S2). Altogether 36 and 10 proteins were found for the Y3 + Y6 and Y6 + Y9 groups, respectively, and showed uniformly increased or decreased expression levels (Fig. 2). Sixteen proteins with altered expression patterns at all three time points were identified (APMAP, CPT2, DECR1, FABP4, FABP5, GDI2, HMMR, KRT4, KRT13, KRT15, LGALS7, MLEC, PTH, RAP1A, TKT, and TUFM; Fig. 3).

In plasma from irradiated young rats, 328 proteins were identified, of which 50, 123, and 66 proteins were unique in the Y3, Y6, and Y9 groups, respectively (Fig. 1c, Supplementary Table S4). Twenty proteins with highest differential expression levels are presented in Fig. 2. None of these proteins was associated with thyroid function according to HPA. In total, 77 proteins were present in two of the three groups, and the proteins with uniformly increased or decreased expression levels for Y3 + Y6 and Y6 + Y9 groups are presented in Fig. 2. Twelve differentially expressed proteins were detected in all groups (CA1, CAPN1, eEF1A1, F7, FTL1, GPD1, PF4, PLEC, PKLR, PROC, PVALB, and RGS18; Fig. 3). The CA1 protein had uniformly increased expression level, and the FTL1 and PVALB proteins revealed uniformly decreased expression levels for all three groups.

### Time-related expression profiles in irradiated versus non-irradiated adult rats

In total, 456 differentially regulated transcripts (corresponding to 426 genes) were identified in the irradiated thyroid tissue of adult rats using RNA microarray analysis, with 16, 18 and 412 RNA transcripts (16, 18 and 382 genes) uniquely detected in the A3, A6, and A9 groups, respectively (Fig. 1d, Supplementary Table S5). Twenty RNA transcripts with the highest regulation are presented in Fig. 2. According to HPA, the *Tg* (A3) and *Marveld2*, *Sorcs1*, *Ipcef1* and *Irs4* (A9) genes were thyroid-related (Supplementary Table S2). Only the *Vegfb* transcript was significantly regulated in all adult groups (A3, A6, and A9). However, *Vegfb* was up-regulated in the A3 and A6 groups and down-regulated in the A9 group (Fig. 3).The *Vegfb* and *Klk1c9* transcripts were uniformly expressed in the A3 + A6 group (Fig. 2).

A total of 806 differentially expressed proteins were detected in adult thyroid tissue using LQ-MS/MS analysis, with 444, 142 and 43 unique proteins identified in the A3, A6 and A9 groups, respectively (Fig. 1e, Supplementary Table S6). The 20 proteins with highest expression levels are presented in Fig. 2. The OCLN (A3) was the only protein associated with thyroid function according to HPA (Supplementary Table S2). Only two of the 165 proteins detected in two groups were thyroid-specific, i.e. ACADL (A3 + A6) and TG (A6 + A9) (Supplementary Table S2). Among the identified proteins with uniform expression, nineteen were found in the A3 + A6 groups and six in the A6 + A9 groups (Fig. 2). Totally, 12 proteins (CPQ, DPYSL2, EIF3J, GOT1, HSPB6, HMGN2, KRT1, KRT13, MYBPC1, PTH, PVALB, and RPLP2) were detected in all three adult groups (Fig. 3). The HSPB6 protein showed increased expression levels for all groups in adult rat thyroid tissue.

The LQ-MS/MS analysis identified 375 differentially expressed proteins in plasma from irradiated adult rats, of which 70, 102, and 82 proteins were unique for the A3, A6, and A9 groups, respectively (Fig. 1f, Supplementary Table S7). The 20 proteins with the highest differential expression levels are presented in Fig. 2. None of these proteins was associated with thyroid tissue according to HPA. In total, 109 regulated proteins were identified in two of the groups, where 19 proteins with uniform expression were found in the A3 + A6 groups and two in the A6 + A9 groups (Fig. 2). Twelve regulated proteins were commonly detected in all adult groups (HIST1H1E, MCPT1, FN1, HINT1, APOC4, eEFEA1, HIST1H4B, HSP90B1, F9, MYL6, LBP, and FKBP1A; Fig. 3).

### Common effects irrespective of age and time

sixteen regulated RNA transcripts (16 genes) in thyroid, and 174 and 101 proteins in thyroid and plasma, respectively, were found in both young and adult rats for at least one of the three time points after ^131^I injection (Fig. 1g–i). Two proteins in thyroid (KRT13 and PTH) and one protein in plasma (eEF1A1), but no RNA transcript in thyroid were identified at all time points (Fig. 3). KRT13 protein expression was increased in the Y6, Y9, A3, and A6 groups and decreased in the Y3 and A9 groups. PTH protein expression was elevated in the Y9 and A9 groups and reduced in the remaining four groups. The expression of eEF1A1 was increased for Y3, Y9, and A9 and decreased for Y6, A3, and A6 (Fig. 3).

Of the 20 most differentially expressed RNA transcripts and proteins in rats irrespective of age the *Pth* and *Krt13* genes and the PTH, and KRT13 proteins were found in thyroid tissue for at least one time point (Supplementary Tables S8–Tables S10).

### Age-related effects

**Figure 4:**
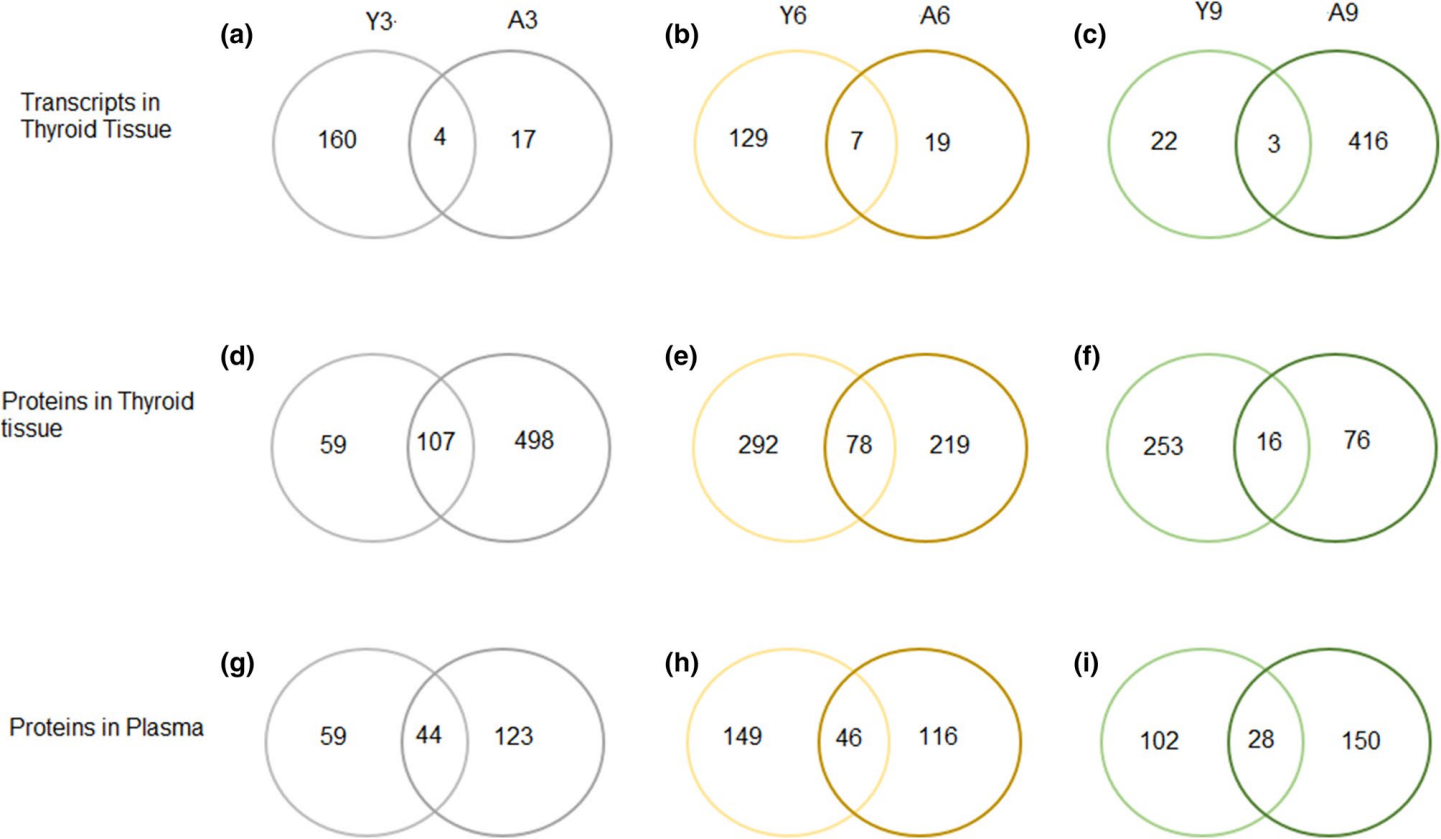
Venn diagram of age-related transcripts in thyroid and proteins in thyroid and plasma. Panels (**a**–**c**) display common and unique transcripts in thyroid for Y3 *vs.* A3, Y6 *vs.* A6 and Y9 *vs.* A9, respectively. (**d**–**f**) displays common and unique proteins in thyroid for Y3 *vs.* A3, Y6 *vs.* A6 and Y9 *vs.* A9, respectively. (**g**–**i**) displays common and unique proteins in plasma for Y3 *vs.* A3, Y6 *vs.* A6 and Y9 *vs.* A9, respectively. Y represents young rats and A represents adult rats, and the following numbers indicates the number of months after ^131^I administration.

In total 181, 155, and 441 RNA transcripts were found differentially expressed when comparing young and adult rats 3, 6 and 9 months after ^131^I exposure (Fig. 4a–c), of which 160 and 17, 129 and 19, and 22 and 416 transcripts were uniquely regulated in Y3 and A3, Y6 and A6, and Y9 and A9 groups, respectively. In total, 167, 589 and 345 proteins in thyroid tissue were identified after 3, 6 and 9 months, respectively (Fig. 4d–f), including 59 and 498, 292 and 219, and 253 and 76 uniquely regulated proteins in Y3 and A3, Y6 and A6, and Y9 and A9 groups, respectively. In total, 227, 311 and 280 proteins in plasma were identified after 3, 6 and 9 months, respectively (Fig. 4g–i), of which 59 and 123, 149 and 116, and 102 and 150 were uniquely regulated in Y3 and A3, Y6 and A6, and Y9 and A9 groups, respectively.

### Time-related effects irrespective of age

**Figure 5:**
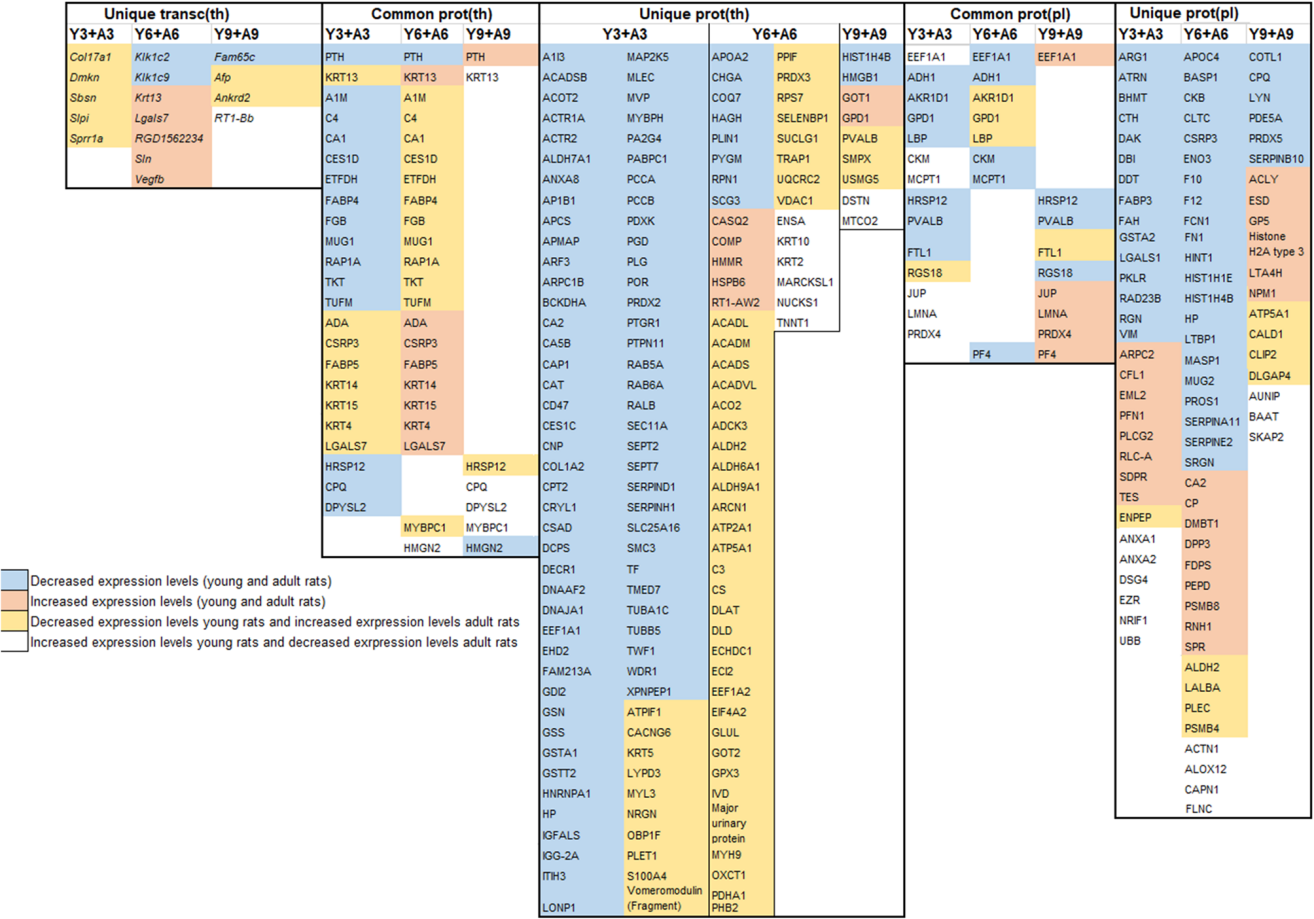
Transcripts and proteins differentially regulated in both young and adult rats three, six and nine months after ^131^I injection. Data are presented separately for common proteins (differentially regulated at two or three time-points) and unique transcripts or proteins (differentially regulated at one time-point only). Blue colour represents decreased and red colour increased expression level, yellow represents decreased expression in young and increased expression in adults, white represents decreased expression in young and increased expression in adult individuals.

The 16 regulated RNA transcripts (16 genes) in thyroid were unique for only one time point; 5 (Col17a1, *Dmkn*, *Sbsn*, *Slpi* and *Sprr1a*), 7 (*Klk1c2*, *Klk1c9*, *Krt13*, *Lgals7*, *RGD1562234*, *Sln*, and *Vegfb*) and 4 (*Ankrd2*, *Afp*, *Fam65c* and *Rt1-Bb*) at 3, 6 and 9 months, respectively, when evaluating data for young and adult rats together (Figs. 1g, and 5). The number of regulated unique proteins in thyroid for each time point were 84, 56, and 9 after 3, 6 and 9 months, respectively (Figs. 1h and 5). Corresponding numbers of unique proteins in plasma were 30, 37 and 19 (Figs. 1i and 5). Moreover, KRT13 and PTH proteins in thyroid and eEF1A1 protein in plasma were identified in all age groups at the three time points (Fig. 3).

In a separate analysis at each time point, five, seven and four transcripts were in common for Y3 + A3, Y6 + A6 and Y9 + A9 groups, respectively (Fig. 4). Furthermore, 107, 78 and 16 proteins were in common for Y3 + A3, Y6 + A6 and Y9 + A9 groups, respectively. The number of proteins in common for Y3 + A3, Y6 + A6 and Y9 + A9 groups were 44, 46 and 28, respectively.

### Hormone levels in plasma

Nine months after exposure the plasma levels of T3 was about twice higher in exposed rats (162 ± 1(SD) in young and 166 ± 2(SD) in adult rats) compared with controls (80 ± 1(SD) and 79 ± 3(SD), respectively).

Corresponding values for T4 were 43 ± 1(SD), 42 ± 3(SD), 39 ± 5(SD) and 46 ± 2(SD), respectively. The TSH level in all samples were lower than the detection limit of the kit.

### Histological evaluation of rat thyroid tissue

Individual thyroid tissue samples were morphologically evaluated for each rat. In the young exposed rats 3/6, and in adult exposed rats 1/6 had neoplastic changes, respectively. In the young control group 4/6 and in the adult control group 3/6 had neoplastic changes.

### Pathway analysis using GO term annotation and IPA software

#### Significantly enriched GO terms included metabolism and cellular integrity

**Figure 6:**
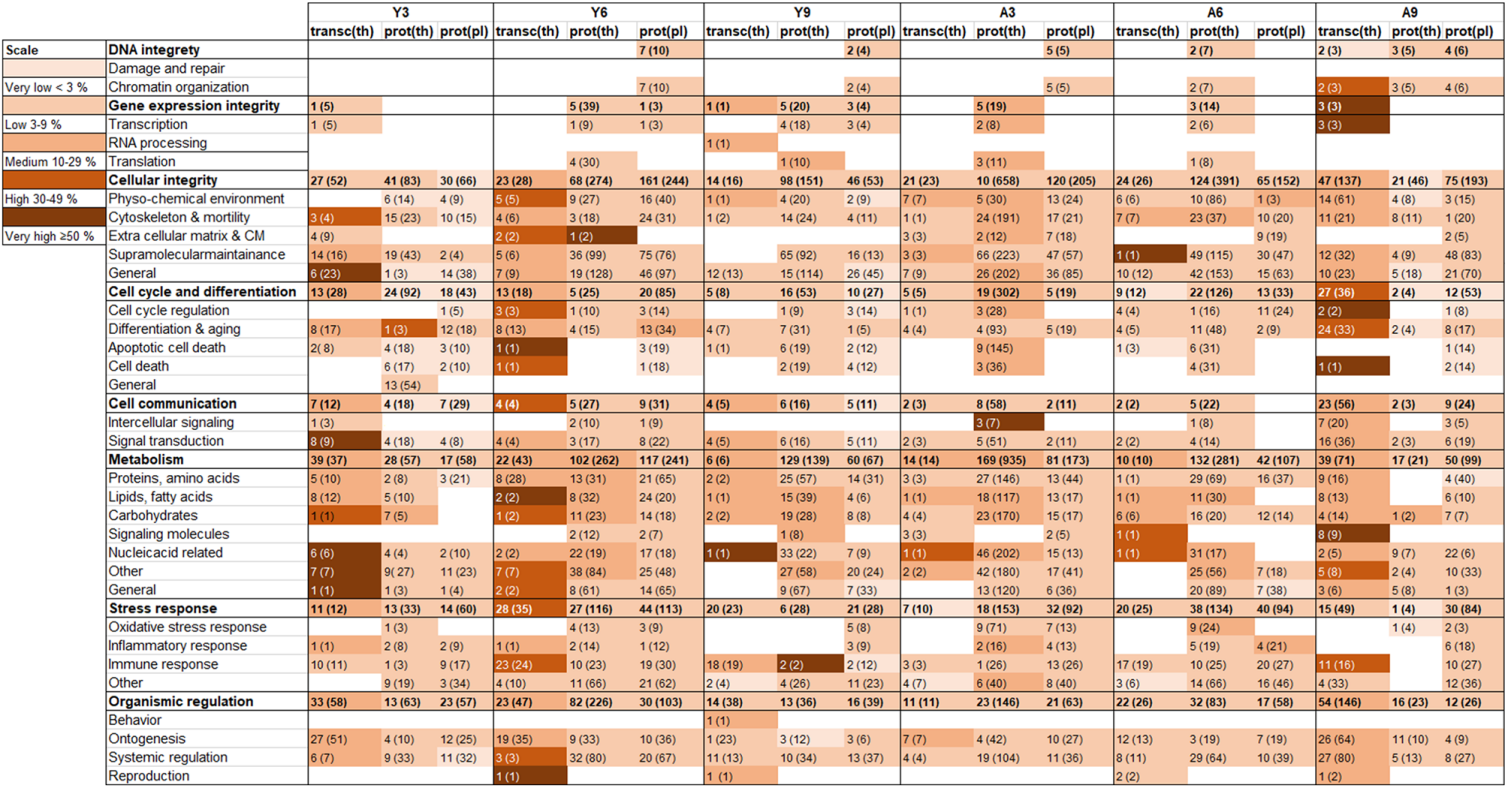
Heat map of transcript and protein response in thyroid tissue and plasma, showing enriched biological processes categorised by GO terms for cellular function. The heat map was constructed from transcripts in thyroid transc(th) and proteins in thyroid prot(th) and proteins in plasma prot(pl) with significant levels by using categorisation of enriched biological processes based on GO ancestor charts. The percentage of scored vs filtered transcripts or proteins is displayed by very light orange, light orange, orange, dark orange and brown representing the percentage very low < 3%, low 3–9%, medium 10–29%, high 30–49% and very high ≥ 50%, respectively. The number of scored transcripts or proteins are displayed, and the total number of associated GO terms are presented in parentheses. Y = young, A = adult.

The distribution of enriched GO terms in the 18 groups were evaluated and displayed in a heat map (Fig. 6). Overrepresented GO terms included cell cycle and differentiation and metabolism, while DNA integrity and gene expression integrity were detected to a lower extent. Stress response and cell communication were enriched in the transcriptomic data, but less so in the proteomic data (thyroid and plasma). The associated GO terms for the 16 commonly regulated proteins in young rat thyroids were mainly associated with metabolism, signal transduction, physiochemical environment, and cell death. The 12 thyroid tissue proteins obtained in adult rats were primarily associated with metabolism, oxidative stress, immune system, inflammation, differentiation and aging. For the 12 significant proteins in plasma for young rats, associated GO terms included cytoskeleton & motility, general and protein metabolism, RNA processing, signal transduction, and supramolecular maintenance. The GO terms for the corresponding 12 proteins in plasma from adults included intercellular signalling, RNA processing, signal transduction, supramolecular maintenance and transcription. Several proteins were commonly detected in both young and adult rats. The PTH associated GO terms included e.g. metabolism, proteins and signal transduction, while cytoskeleton and motility, ontogenesis, also stress response and supramolecular maintenance were related to KRT13, and cell death and apoptosis for eEF1A1. Furthermore, the *Vegfb* transcript was only detected in adult rats and was associated with several GO terms, e.g. apoptotic cell death, cell cycle regulation and immune response.

### Up-stream regulator analysis with IPA software

Significantly expressed RNA transcripts and proteins were used for a group-wise analysis of canonical pathways and up-stream regulators to evaluate radiation-induced cellular mechanisms detected in thyroid tissue and plasma (Supplementary Table S11 and Table 1). The majority of the canonical pathways were detected in one group. Some canonical pathways were more prevalent than others, e.g. Ephrin signalling (down-regulated Y3, Y6, A3, and up-regulated in Y9), Integrin signalling (up-regulated for Y9 and A3, and down-regulated for Y3, Y6 and A3) and Paxillin signalling (up-regulated Y6, Y9, A3 and A6). RhoA related signalling pathways were seen primarily in young individuals, but also for the A3 group (up-regulated for all except for two pathways in Y3). Only three upstream regulators were detected (Table 1). PPARG was identified as an upstream regulator for Y6 and A3 (down-regulated), and for Y9 and A6 (up-regulated) groups. In addition, VCAN was present for, Y6 and A9 (up-regulated), and A6 (down-regulated). NFkB was identified in the Y9 group.

**Table 1:** Upstream regulators for ^131^I exposure in young and adult rats from the IPA software analysis. Activated (z > 2.0) or inhibited (z < − 2.0) signalling pathways, are presented.

Upstream regulators	p	z	Target molecules in dataset
**Y6**			
Proteins in thyroid			
PPARG	5.2 × 10^–14^	− 3.1	ACADL, ACADS, ACLY, ACSL1, CD36, CS, DLAT, FABP4, HADHA, HADHB, PC, Slc25a1, SLC25A20, TAGLN
Proteins in plasma			
VCAN	6.4 × 10^–3^	2.2	APOE, LBP, RNASE4, TGFB1, VCL
**Y9**			
Proteins in thyroid			
PPARG	4.8 × 10^–10^	3.1	FABP5, KRT19, Ldha/RGD1562690, SOD2
NFkB (complex)	3.6 × 10^–2^	2.0	COL1A1
**A3**			
Proteins in thyroid			
PPARG	2.8 × 10^–12^	− 3.8	ACADL, ACADS, ACLY, ACSL1, CD36, CS, DLAT, FABP4, HADHA, HADHB, MDH1, MGLL, PC, Slc25a1, SLC25A20
**A6**			
Proteins in thyroid			
PPARG	6.3 × 10^–5^	3.0	ACADL, ACADS, CS, DLAT, FABP4, MDH1
Proteins in plasma			
VCAN	4.4 × 10^–4^	− 2.4	ASS1, C1S, ICAM1, LBP, LGALS3, PAM
**A9**			
Proteins in plasma			
VCAN	2.3 × 10^–2^	2.0	LBP, MSLN, PRDX5, RNASE4

## Discussion

The current work was designed to expose young and adult rats with ^131^I in order to obtain an absorbed dose to the thyroid of 100 mGy, which is in the same range as children and adults received due to the Chernobyl accident. We also chose to relate our findings not only with thyroid cancer, but also effects on the thyroid function.

In the present study, radiation-induced effects on the proteome and transcriptome expression patterns in thyroid and plasma varied depending on the age of exposed rats and time after exposure. No general trend in differences in number of differentially expressed RNA transcripts and proteins was found between adult and young rats or with time after exposure. Four potential age- and time-dependent biomarkers showed unidirectional expression (up- or down-regulation) levels for all three time points, including three in young rats (CA1, FTL1, and PVALB proteins in plasma) and in adults one protein in thyroid tissue (HSPB6). Of these, only CA1 varied monotonously with time. Three biomarker candidates, depending on time and independent of age, were found, however, with varying direction of expression: two proteins in thyroid tissue (KRT13 and PTH) and one protein in plasma (eEF1A1). The PTH protein had either decreased (3 and 6 months) or increased (9 month) expression levels for both young and adult individuals at the same time point. No single biomarker candidate related to age irrespective of time after exposure was identified. Consequently, a panel of 20 proteins with the highest up-regulation in plasma, related to age and irrespective of time after exposure, is proposed for each age group (young and adult) (Table 2). The *Vegfb* transcript was identified in adults at 3 (up-regulated), 6 (up-regulated), and 9 months (down-regulated) after exposure and is suggested as a biomarker candidate that is related to thyroid function. Furthermore, several of the identified transcripts and proteins, correlated to age at a certain time-point, have previously been related to thyroid diseases. Of these, *Ada* (Y3), *Tgm3* (Y3 and Y6), *Vegfb* (A3 and A9) and *Pth* (A9) genes in thyroid tissue, RPN2 (Y9 and A3), DLD (A3) and ACADL (A3) in thyroid tissue and PVALB (Y6) in plasma were previously proposed as long-term biomarker candidates in our previous study^24^.

**Table 2:** Panels of biomarker candidates in plasma for ^131^I exposure of young and adult individuals, respectively. The panel consists of the 20 proteins with the highest increased expression for each age group.

Panel for young individuals				Panel for adult individuals			
Proteins	Y3	Y6	Y9	Proteins	A3	A6	A9
ACTA2		×		ANXA1			×
ACTB		×		CMPK1			×
ANXA1	×			DHTKD1		×	
CA1	×	×	×	EEF1A1			×
CA2		×	×	EZR			×
COPS4			×	HIST1H1E			×
CORO1A		×		HIST1H2BA			×
CPS1			x	Histone H2A type 3			×
DSG4	×		×	Histone H3.1			×
LGALS5			×	HNRNPA2B1			×
NPM1	×		×	HNRNPC			×
NRIF1	×			HNRNPK			×
PFN1	×	×		LMNA			×
PRDX4	×		×	NPM1			×
PSBPC2		×		NRIF1			×
RGS18		×		PGAM1			×
RT1-AW2	×	×		PIGR		×	
SPRR1A	×			PPP1R7		×	
TPM4		×		PRDX4			×
XK	×			VCP			×

In the present investigation, several types of biomarkers could be identified: (a) biomarkers dependent on age and time after exposure, (b) time-dependent biomarkers independent of age (c) age-dependent biomarkers but independent of time after exposure, and (d) biomarkers independent of age and time after exposure (related to exposure or not). Ideally, biomarker candidates should be related to effects on thyroid tissue e.g. thyroid function and thyroid carcinogenesis. Furthermore, biomarker candidates with uniformly increased or decreased expression levels are technically easier to evaluate at any time point. It is also important that the biomarkers have relatively high differential expression as an effect of irradiation compared with noise due to small individual differences. When no single RNA transcript or protein can reflect a defined property (such as absorbed dose or time after exposure), a panel of biomarkers with expression patterns depending on these properties may be more appropriate^25^. An optimal biomarker panel should include up-regulated markers, to be able to easily distinguish exposed from non-exposed individuals. It would also be preferable if the biomarker candidates are detectable in blood samples, which are less invasive than tissue biopsies.

Four age- and time-dependent biomarker candidates with unidirectional expression were proposed, i.e. the CA1 (up-regulated), FTL1 (down-regulated), and PVALB (down-regulated) proteins were identified in plasma for young rats, and the HSPB6 (up-regulated) protein was identified in adult rat thyroid tissue. The CA1 is an enzyme involved in several biological processes e.g. respiration, bone resorption, saliva and gastric acid^26^. A previous study found low CA1 levels in erythrocytes from hyperthyroid patients and proposed CA1 as a biomarker for different types of hyperthyroidism^27^. The HSPB6 small heat shock protein maintains denatured proteins in a folding compartment state, and is involved in smooth muscle relaxation^26^. The FTL1 protein is involved in iron storage in various tissues^26^. The PVALB calcium ion-binding protein was previously proposed as a radiation biomarker candidate^26,28^.

The KRT13, PTH, and eEF1A1 proteins were identified at all time points for young and adult individuals and were proposed as time-dependent, but age-independent biomarker candidates. The keratin (*KRT*) gene family encodes intermediate filaments involved in the internal cellular structure stabilisation and maintenance of cell shape^29^. The parathyroid hormone (PTH) expression levels were consistent with time after exposure (three (up-regulated), six (up-regulated) and nine months (down-regulated)) for both young and adults. PTH regulates calcium and metabolism^26^. *Krt13* (Y3 and A6) and *Pth* (A9) were identified among the 20 transcripts with the highest expression (Supplementary Table S8). The eukaryotic translation elongation factor A1 (eEF1A1) is a GTP-binding protein involved in cell growth, signal transduction, differentiation and apoptosis. Elevated eEF1A1 expression levels have been associated with increased cell proliferation and oncogenic transformation, and overexpression of mRNA was correlated with metastasis^30^. Previously, the eEF1A1 protein was proposed as a biomarker for cellular senescence after radiation exposure in cancer cell lines^31^.

In the present study, no single age-dependent but time-independent biomarker candidate was found. Therefore, panels consisting of the top 20 differentially regulated proteins in plasma for young and adult rats are proposed. For young individuals, the panel consists of ACTA2, ACTB, ANXA1, CA1, CA2, COPS4, CORO1A, CPS1, DSG4, LGALS5, NPM1, NRIF1, PFN1, PRDX4, PSBPC2, RGS18, RT1-AW2, SPRR1A, TPM4, and XK. The corresponding panel for adults contains ANXA1, CMPK1, DHTKD1, EEF1A1, EZR, HIST1H1E, HIST1H2BA, Histone H2A type 3, Histone H3.1, HNRNPA2B1, HNRNPC, HNRNPK, LMNA, NPM1, NRIF1, PGAM1, PIGR, PPP1R7, PRDX4, and VCP. Several of these biomarker candidates have previously been associated with thyroid diseases. Positive ACTA2 staining is common in PTCs from adults (27–54 years) and related to higher tumour grade and metastatic potential^32^. High CA1 expression is related to hyperthyroidism, as already discussed. Although *CA2* gene expression was increased in our study, a previous study found decreased *CA2* gene expression in young patients with PTC (15–39 years) but not in older PTC patients (> 40 years)^33^. The methylation regulator HNRNPC was highly expressed in PTC and suggested as part of a molecular signature for PTC progression^34^. The LMNA protein was one of the protein markers identified in a cluster for malignant thyroid cancer^35^. The RT1-AW2 protein is involved in autoimmune thyroiditis^26^. VCP is associated with the sodium iodine symporter (NIS), which is important for normal iodide transport into thyrocytes. Increased VCP expression is related to more aggressive cancer types and reduced iodide uptake, and VCP was suggested as a prognostic biomarker for FTC^36,37^.

The vascular endothelial growth factor B (*Vegfb)* gene was the most interesting single biomarker candidate, related to thyroid function found in adults, and was up-regulated at 3 and 6 months and down-regulated at 9 months after exposure. VEGF plays a pivotal role in angiogenesis^26^. Previously, *Vegfb* was also suggested as a biomarker for ^131^I exposure in mice^23^. To be an ideal biomarker candidate for thyroid function and cancer induction, the RNA transcript or protein should be thyroid tissue related. The *Vegfb* gene is not thyroid tissue related, but TG protein is thyroid-specific and ACADL is involved in metabolic processes, and both were detected in young and adults. However, they were only detected in some of the groups and are therefore not proposed as biomarker candidates in the present study. Taken together, these findings indicate that irradiation has an effect on thyroid function, although effects may vary depending on age and time after exposure.

In the present study, the number of identified GO terms was highest for proteins in thyroid followed by proteins in plasma and RNA transcripts in thyroid. The RNA transcripts showed high correlation to associated GO terms. The enriched GO terms were metabolism and cellular integrity, with the subgroups nucleic acid related metabolism, and cytoskeleton and motility, respectively. High association with metabolism is interesting, since the thyroid hormones triiodothyronine (T3) and thyroxine (T4) play a major part in various metabolic processes. During hyperthyroidism, metabolism in the body is increased via increased T3 and T4 blood levels that results in reduced thyroid stimulating hormone (TSH) levels, and the opposite effect is seen for hypothyroidism^38^. In a study on patients previously treated by external radiation therapy in the neck region (> 40 Gy), nearly half of the patients developed hypothyroidism within 9 months^39^. In the present study T3 levels in plasma from exposed rats were twice higher, irrespective of age, than in corresponding controls after 9 months. The T4 levels were similar in all groups, while the TSH levels were low, indicating increased thyroid hormone production. In general, thyroid cancer usually do not give altered hormone levels. However, the histological analysis of the rat thyroids after 12 months demonstrated neoplastic cells in about half of the young animals and only one of the exposed adult rats. However, neoplastic cells were also found in some unexposed rats.

Canonical pathways related to signal transduction (Ephrin receptor signalling, Paxillin signalling, and integrin signalling) were predominantly seen in the young groups and A3 group. More specifically, the Ephrin receptor is involved in signal transduction and actin cytoskeleton, which controls cellular shape, adhesion and movement by regulation of the Rho GTPase family, including RhoA^40^. Paxillin is a signal transduction adaptor protein that plays a crucial role in plasma membrane-associated adhesion and growth factor signalling, and regulating the actin cytoskeleton^41^. Integrin signalling is involved in the maintenance of the extracellular matrix and in the regulation of the cell cycle by activating several growth-promoting signalling pathways^42^. These findings suggest that radiation can induce cellular activation at low doses shortly after onset of exposure and is sustained over a period of time in young individuals, while the effect decreases with time in adult rats.

IPA analysis identified the upstream regulator PPARG in the majority of the groups (Y6, Y9, A3, and A6). PPARG belongs to the nuclear receptor family of transcription factors and is expressed at very low amounts in normal thyroid. PPARG is also involved in, e.g., regulation of tumour growth in several cancer forms^26^. Elevated *PPARG* expression has been observed in PTC tissue^43^. It is also commonly found as an oncoprotein PAX8/PPARG complex in FTC and are more prevalent in younger patients, and PAX8 and PPARG rearrangements may be important in PTC development^36,37^. The transcription factor NFkB is known to be activated after radiation exposure, which may result in cancer development^44^. Interestingly, NFkB was activated in the Y9 group only indicating a higher risk to induce cancer in young individuals.

No RNA transcript was in common for all young rats in the present study. This finding can have several explanations. One is that total RNA microarray analyses were performed on individual samples, while protein analyses used pooled protein samples for each group. Furthermore, the transcriptional response might be more rapid and/or inconsistent over time, and transcripts are frequently degraded quickly, while proteins are more stable over time. *Dbp* (Y9), was the only transcript identified in the present study as well as in our previous work^22,28^. We also identified the following proteins both in the present study and in similar previous short-term studies on mice; in thyroid tissue: AOC3 (A3), ATP2A1 (Y6, Y9, A3, and A6), CPA3 (A3), PVALB (Y9, A3, A6 and A9), S100a8 (A3), S100a9 (A3), TNNI2 (Y9, A6), and TNNT3 (A6) and PTH (all groups), ACADL (Y6, A6; and in plasma: PVALB (Y3, Y6, Y9 and A3) and ENO3 (A6)^20,21,23,28,45,46^. However, our former studies were short-term studies and some used another radionuclide, which might explain differences in results. In a previous review summarising more than 300 publications, 261 radiation responsive proteins were proposed^47^. Of these proteins, 33 were also identified in the present study, including AFP, ALDH2, APOA1, APRT, BID, BLVRA CA1, CA2, CALR, CKM, CSTB, DDT, FABP5, GSS, FTH1, GGT1, GLO1, GPT, GPX1, HP, ICAM1, ILK, LYN, NPM1, PRDX2, PRDX3, PRDX5, PRDX6, PSMB4, SERPINB10, SPTAN1, TF, and VIM.

In comparison with adults, ^131^I irradiation of the thyroid is expected to have a profound effect on children, since children are more sensitive to radiation and showed higher incidence of thyroid cancer after the Chernobyl accident. RNA microarray analyses of PTC tumours from primarily children exposed to radioactive fallout from the Chernobyl accident resulted in a plethora of suggested biomarkers, potentially due to the difficulty of using a representative control group (regarding age, dietary, geographical area, gender, etc.)^9–15,48–58^. Furthermore, CLIP2 and TG were the only previously suggested biomarkers that were also identified in the present study^11,12,16,53,54^. The CLIP2 protein was detected in plasma in the nine month groups, indicating that the time of exposure to the endpoint has a profound effect on whether certain genes or proteins can be detected. In contrast, the TG protein was differentially expressed in several of the groups, which is interesting since TG is involved in thyroid hormone production^19^.

In conclusion, the present study showed that radiation-induced effects depended on age, e.g. a higher number of regulated proteins were found in tissues from adult rats. Moreover, time after exposure affected the transcriptomic and proteomic response, although no general trend was identified. Promising biomarkers were age- and time-dependent in young rats (CA1, FTL1 and PVALB, plasma) as well as in adult rats (HSPB6, thyroid tissue). The ^131^I exposure biomarker candidate (time-dependent but age-independent), PTH (in thyroid tissue), was detected in all six groups. Panels of biomarker candidates (age-dependent and time-independent) consisting of 20 proteins in plasma for young and adult rats, respectively, were proposed. Several of the identified biomarkers were among the previously identified specific genes involved in thyroid cancer and function. Furthermore, *Vegfb* (adults) was identified as a thyroid function biomarker candidate*.* However, these proposed biomarker candidates need to be further validated in human samples as a step towards clinical application or for radiation protection purposes. Altogether, none of these proposed transcripts or proteins can be considered as radiation-specific.

## Methods

### Experimental set-up

Forty healthy Sprague Dawley rats (Taconic, Denmark) were randomly divided into ten groups containing four individuals each. Rats in six groups were i.v. injected with 50 kBq ^131^I: rats in 3/6 groups were young, injected at 5 weeks of age, and the remaining 3/6 groups rats were adult, injected at seventeen weeks of age. The estimated absorbed dose to the thyroid was 0.1 Gy and 0.07 Gy for young and adult rats, respectively, assuming similar percentage uptake but smaller thyroid in young rats^59,60^. The remaining four groups were age-matched untreated controls.

The animals were killed under pentobarbital (APL; Kungens kurva, Sweden) anaesthesia at three, six or nine months after study start. The number of control groups were optimised by simultaneously terminating the 3 months adult rats and the 6 months young rats, and the 6 months adult rats and the 9 months young rats, respectively. Individual thyroid samples were collected, flash-frozen in liquid nitrogen, and stored at − 80 °C until further analysis. Plasma samples were separated from individual blood samples, using heparin filled syringes and then flash-frozen in liquid nitrogen and stored at − 80 °C.

The animals were under daily supervision and had free access to standard rat chew and water. We did not record any weight loss or impaired general health condition in any of the rats. The experimental set-up was approved by the Ethical Committee on Animal Experiments in Gothenburg, Sweden (Permit Number: 146-2015). All methods were performed in accordance with the relevant guidelines and regulations and are reported in accordance with ARRIVE.

### Gene expression analysis

Total RNA was extracted from flash-frozen thyroid samples using the RNeasy Lipid Tissue Mini Kit (Qiagen; Hilden, Germany). Microarray analysis was performed at the Bioinformatics and Expression Analysis Core Facility at Karolinska Institute (Stockholm, Sweden) using Agilent Sureprint G3 Rat GE 8 × 60 K microarrays (Agilent, Santa Clara, CA, USA). Nexus Expression 3.0 (BioDiscovery; El Segundo, CA, USA) was used to identify differentially expressed transcripts by comparing data from exposed and non-exposed individuals. The fold change cut-off value was set to 1.5 and the FDR adjusted p-value (Benjamin–Hochberg method) was set to 0.01. Gene Ontology (GO) terms were used for functional annotation. A p-value < 0.05 was compiled with Nexus Expression and associated with different cellular functions using an in-house model as previously described^45^*.* Human Protein Atlas (https://www.proteinatlas.org) was used to identify thyroid-specific genes.

### Tandem mass spectrometry (LC–MS/MS) analysis

Total cellular proteins were extracted from thyroid and plasma samples and labelled with isobaric labels, TMT 10-plex (Thermo Fisher Scientific, Waltham, MA, USA). Thyroid samples for each irradiated group and controls were pooled, respectively. The plasma samples were pooled in the same manner. Tandem mass spectrometry (LC–MS/MS) was performed at the Proteomics Core Facility at University of Gothenburg (Gothenburg, Sweden) as previously described^24^*.* Differentially expressed proteins were identified at ≥ 1.5-fold change. The (DAVID) bioinformatics resource tool (https://david.ncifcrf.gov/) was used for functional protein annotation. Proteins were associated with GO terms and categorised in a similar manner as the RNA microarray data. Human Protein Atlas (https://www.proteinatlas.org) was used to identify thyroid-specific proteins.

### Ingenuity pathway analysis (IPA)

Ingenuity pathway analysis (IPA) software (Ingenuity Systems, Redwood City, USA) was used to analyse affected canonical pathways, biological functions and upstream regulators for the identified transcripts and proteins. Fisher’s exact test (p < 0.05) was used for statistical significance analyses. Upstream regulators with z-score > 2 or z-score <  − 2 were denoted as activated or inhibited, respectively.

### Hormonal assays

Thyroid hormone levels were measured in plasma from young and adult rats, 9 months after exposure, and from corresponding controls. The plasma concentration of thyroid stimulating hormone (TSH) and triiodothyronine (T3) were measured using ELISA kits (CSB-E05085r, CSB-E05115r, Cusabio, Houston, TX, USA). Thyroxine (T4) levels were measured using ELISA kit (GWB-39U2L8, GenWay, San Diego, CA, USA). All ELISA plates were read on Victor 31420 multilabel plate counter (Perkin Elmer, Waltham, MA, USA).

### Histological analysis of thyroid tissue

In a group of other rats, treated in a similar way, thyroid tissue was surgically removed from each rat after twelve months. Thyroid tissue was fixed in formalin and imbedded in paraffin for histological analysis. A certified pathologist evaluated thyroid tissue morphology, abnormal tissue structure, and the presence of tumour cells, using paraffin sections (4 µm) stained with haematoxylin and eosin.

## Supplementary Information

Supplementary Tables.

## Data Availability

The mRNA datasets created and analysed are available at NCBI’s Gene Expression Omnibus^61^ and are accessible through GEO Series accession number GSE146051 https://www.ncbi.nlm.nih.gov/geo/query/acc.cgi?acc=GSE146051. The mass spectrometry proteomics data have been deposited to the ProteomeXchange Consortium via the PRIDE^62^ partner repository with the dataset identifier PXD017715. The data for the Y9 group has been published elsewhere^24^.
