# Supplementary material for: Age-related long-term response in rat thyroid tissue and plasma after internal low dose exposure to 131I
Source: Sci Rep. 2022 Feb 8;12:2107. doi: 10.1038/s41598-022-06071-4 (PMC8825795; doi:10.1038/s41598-022-06071-4)
Supplement: Supplementary file 1 — Supplementary Tables. [file 41598_2022_6071_MOESM1_ESM.pdf]

S Table 1 ***Unique regulated thyroid transcripts for each test group with a fold change value above 1.5 or under -1.5 for young rats killed three, six and nine month after*** ^{131}***I administration respectively.***

Thyroid transcripts young rats							
**Y3**		**Y3 cont.**		**Y6**		**Y9**	
**Gene**	**Fold Change**	**Gene**	**Fold Change**	**Gene**	**Fold Change**	**Gene**	**Fold Change**
A4galt	-3.73	*Perp*	-2.94	*Abat*	-2.58	*Acan*	2.55
Abca12	-3.88	*Pkp3*	-3.86	*Adh7*	4.04	*Adra1b*	-1.91
Ada	-4.15	*Plekhf2*	2.41	*Asprv1*	7.56	*Afp*	-3.86
Alox12e	-5.37	*Pof1b*	-5.71	*Bpifa5*	3.34	*Ankrd2*	-2.09
Aloxe3	-3.34	*Prdm1*	-3.47	*Car7*	2.60	*Bpifa1*	2.35
Ankrd22	-3.28	*Pth*	-3.17	*Col23a1*	-2.01	*Cd300lg*	2.19
Anxa9	-4.35	*Rhcg*	-5.50	*Cpa4*	2.57	*Ceacam16*	-1.34
Bnipl	-3.85	*Rnf39*	-3.95	*Dcdc2*	2.66	*Chad*	2.52
Casr	-3.91	*RT1-M4*	3.54	*Ect2*	1.96	*Col10a1*	4.54
Cdhr1	-3.52	*Sdcbp2*	-3.47	*Epyc*	2.64	*Col10a1*	4.61
Ces1e	-3.43	*Sdr16c5*	-3.47	*Fam163a*	2.92	*Col2a1*	1.81
Chga	-4.62	*Serpina9*	-3.59	*Fam25a*	6.07	*Col9a2*	1.47
Clca5	-3.59	*Serpinb11*	-3.86	*Fetub*	2.59	*Dbp*	-1.81
Cryba2	-5.50	*Serpinb13*	-4.00	*Il1r2*	1.98	*Entpd2*	-1.72
Cst6	-3.19	*Slc15a1*	-4.26	*Kap*	-3.14	*Fam65c*	-1.78
Cyp2t1	-3.54	*Sostdc1*	-3.87	*Klhl14*	3.16	*Gdf15*	-1.61
Dsc1	-4.10	*Sox21*	-3.80	*Klk12*	2.97	*Glis1*	-1.18
Dsg1	-3.64	*Sptbn2*	-4.31	*Klk1c2*	-2.54	*LOC102546809*	1.30
Dsg1	-3.34	*Tgm1*	-4.65	*Klk1c9*	-4.05	*LOC171161*	2.34
E4f1	-3.05	*Them5*	-4.05	*Krt15*	3.61	*Matn1*	2.78
Elmod1	-3.77	*Tmprss4*	-3.84	*Lce1l*	3.20	*Muc16*	2.63
Fam155b	2.68	*Tnfrsf11b*	-3.06	*Lce1m*	2.38	*RGD1305045*	-2.56
Fam57a	-3.41	*Tns4*	-4.29	*Lilrb3l*	-3.07	*RT1-Bb*	2.59
Fgfbp1	-3.49	*Tpo*	-2.51	*Lipf*	-3.60	*Scgb1a1*	2.12
Fut1	-4.26	*Trpv5*	3.46	*LOC685560*	-2.76	*Scgb3a1*	2.77
Gcm2	-4.95	*Tspan6*	-2.39	*Mettl21c*	2.15		
Gldc	-3.93	*Uba1y*	-3.34	*Mmrn1*	2.02		
Gpr64	-3.21			*Myh4*	2.65		
Gsdma	-3.73			*Pglyrp3*	2.61		
Gsta2	-4.05			*Pglyrp3b*	3.00		
Hsd17b2	-4.31			*RGD1561341*	-2.35		
Kl	-4.57			*RGD1563692*	3.12		
Klk11	-3.47			*RGD1565410*	3.12		
Krt1	-4.35			*RT1-CE5*	2.97		
Lce1f	-4.30			*Scel*	4.16		
Lipk	-3.57			*Sim2*	3.34		

LOC102551453	-4.00	*Slc4a9*	2.91
LOC102557423	-3.78	*Sln*	2.64
LOC363337	-3.75	*Spata16*	-2.63
LOC500737	3.93	*Spink5*	2.58
Lypd3	-2.78	*Tg*	2.66
Nkpd1	-3.64	*Trex2*	2.89
Ocm2	-4.54	*Trpm6*	3.01
Olr184	3.84	*Vcsa1*	-3.56
Ooep	-3.64	*Vegfb*	5.67
Paqr5	-6.00	*Vsnl1*	2.51

S Table 2. Thyroid associated transcripts and proteins according to Human Protein Atlas. Totally eight transcripts and six proteins were seen in thyroid tissue. No protein were seen in plasma.

Thyroid specific transcripts and proteins		
**Transcript**	**Fold change**	**Group**
Tpo	-2.51	Y3
Klhl14	3.16	Y6
Tg	2.66	Y6
Tg	2.45	A3
Marveld2	-1.43	A9
Sorcs1	-1.18	A9
Ipcef1	-1.53	A9
Irs4	-1.91	A9
**Protein (thyroid tissue)**	**Fold change**	**Group**
OCLN	1.63	Y6
SORBS2	1.64	Y9
ACADL	-2.11/2.49	Y6/Y9
OCLN	1.80	A3
ACADL	-3.17/1.78	A3/A6
TG	-1.95/-2.01	A6/A9

S Table 3 ***Unique regulated thyroid proteins for each test group with a fold change value above 1.5 or under -1.5 for young rats killed three, six and nine month after ^{131}I administration respectively.***

Thyroid protein young rats							
**Y3**		**Y6**		**Y6 cont.**		**Y9**	
**Protein**	**Fold change**	**Protein**	**Fold change**	**Protein**	**Fold change**	**Protein**	**Fold change**
ACPP	-1.63	ACO1	-3.10	PRKACA	-1.94	AARS	1.55
ACTC1	-1.81	ACSL1	-3.29	PSAP	1.55	ACAN	1.69
ACTR1A	-1.57	ACY3	-1.59	PSBPC2	15.18	ACBD5	-1.57
ACTR2	-1.68	ADCK3	-1.56	PSMD1	-1.92	ACTB	1.63
AKT1	-1.91	ADD2	-1.55	PTBP1	-1.86	AGRN	-1.53
ALDH3A1	-4.50	ADH5	-1.84	PTMA	1.75	AKR1A1	1.51
ALDH7A1	-1.70	AHCY	-2.09	PTMS	1.59	AKR1B1	1.63
ANXA8	-3.98	AIFM1	-2.53	PYCRL	-1.75	APOE	-1.65
AP2B1	-1.66	AKR1C9	-1.55	PYGB	-2.68	APRT	1.99
APCS	-1.51	ALDH1A1	-1.76	RAB14	-1.59	ARMC10	1.84
ARPC1B	-2.34	AMBP	-1.54	RAB1A	-1.67	ATP5C1	2.18

ATL3	-1.63	AMPD1	-2.28	RETSAT	1.53	ATP5L	1.60
ATPIF1	-1.56	AMPH	-3.09	RGS9	1.77	ATP6V1B2	1.82
CA5B	-1.81	ANPEP	-1.85	RPL10	-1.86	BLOC1S2	-1.59
CACNG6	-3.36	AP2A2	-1.99	RPL10A	-1.86	BST2	-1.67
CANT1	-1.52	APCS	-2.35	RPL17	-1.88	CABP1	1.60
CAPG	-1.97	APLP2	2.07	RPL24	-1.51	CALCOCO1	-1.66
CAT	-1.96	ARF5	-1.51	RPL8	-1.87	CAPZA2	1.76
CD47	-2.03	ARG1	1.87	RPN1	-1.93	CAPZB	1.58
CLEC2D11	-2.18	ARPP19	1.91	RPS16	-1.51	CASQ1	-1.61
CNN1	-2.88	ASL	-1.53	RPS2	-2.14	CCT2	1.78
COL1A2	-1.76	ATIC	-1.69	RPS27L	-1.90	CCT3	1.76
COPB1	-1.90	BCAT2	-1.78	RPS3	-1.67	CD59	-1.64
DCPS	-1.65	BCL2L1	1.56	RPS4X	-2.36	CD99L2	-1.70
DNAJA1	-1.53	BID	1.69	RPS8	-1.71	CDC42	2.09
DNAJB6	-2.16	BPIFA1	2.11	RT1-AW2	3.71	CDH2	-1.83
DNAJC3	-1.64	C3	-2.87	RTN1	-2.12	CDNF	-1.66
DPYSL3	-2.44	CALCA	-1.58	RTN4	-1.91	CHMP4C	-1.54
DYNLL1	-1.51	CCBL2	-1.94	S100A11	1.74	CMSS1	-1.74
EHD2	-1.50	CD1D	1.91	SAFB	1.56	COQ6	2.09
EHD3	-1.61	CD36	-1.71	SCG3	-1.82	CRK	1.64
EIF2B2	-1.54	CD44	1.55	SCG5	-1.93	CSNK2A1	2.21
ENPP1	-1.71	CD63	1.91	SCGN	-2.18	DAB2	-1.55
ESPN	-1.67	CDH23	-1.74	SDHA	-3.52	DDR1	2.35
F12	-1.51	CHCHD4	1.54	SEC13	-1.90	DHRS4	-2.37
F2	-1.50	CHDH	-2.71	SERPINA 10	-1.83	DNAJC5	-1.79
GSN	-1.72	CHGA	-2.13	SGCE	-1.52	DPP3	2.29
GSTA3	-1.53	CHGB	-1.80	SIL1	1.52	DPP7	1.58
GSTA4	-1.64	COPG1	-1.54	SLC25A1	-1.93	DSTN	1.80
GSTM1	-1.53	COQ7	-1.76	SLC25A11	-2.33	ECHS1	1.53
GSTP1	-1.52	CORO1B	-2.25	SLC25A20	-1.92	ECI1	2.53
GSTT2	-1.61	CORO6	-1.55	SLC25A3	-2.20	EEF1G	1.50
HNRNPA2B1	-1.56	COX6A2	-4.83	SLC25A4	-1.91	Ester hydrolase C11orf54 homolog	2.02
HSPH1	-1.74	CP	-1.64	SMAGP	-1.64	EZR	1.68
IGG-2A	-1.59	CRAT	-3.83	SMC1A	-1.55	F3	-1.51
ITGB4	-1.65	CRYAB	1.53	SSR4	-2.29	FASN	4.30
KHSRP	-1.52	CTBS	-1.79	STBD1	-1.80	FH	1.70
KRT18	-1.52	CTSL	1.51	STK3	1.52	GABARAP	-1.67
KRT8	-1.53	CUL5	-1.50	STOML2	-1.52	GAPDH	1.78

MAP2K5	-2.11	DAP	-5.20	SUCLG1	-2.12	GATC	-1.50
MAPK3	-1.55	DCXR	-1.59	SUOX	-1.61	GDA	1.62
NRGN	-2.03	DDOST	-2.01	TAGLN	1.55	GMFB	1.98
PA2G4	-1.66	DDX39B	-1.54	TBCA	1.76	GOT1	1.72
PABPC1	-1.50	DNAH12	-2.85	TCP1	-2.58	GPD1	1.73
PDXK	-1.52	DNAJC27	-3.81	TIMM22	-2.31	GPI	1.73
PF4	-2.77	DOHH	1.64	TIMM8B	1.67	GPNMB	-1.97
PGD	-1.56	DPP4	-1.79	TIMM9	1.55	GPT	1.93
PHLDB1	-1.60	DR1	1.50	TMED5	1.62	GSTZ1	1.56
PLET1	-1.66	DUPD1	1.67	TMEM109	2.05	GYPC	-1.84
PLG	-1.54	ECH1	-1.56	TMX2	-2.18	HDGFRP3	-1.51
POLB	-1.64	ECI2	-1.51	TNNT1	1.64	Histone H2A type 3	-1.52
PPP1CA	-1.84	EIF3B	-1.64	TPPP3	2.02	Histone H2B type 1	-3.49
PPT1	-1.56	EIF3E	-1.51	TRAP1	-1.82	HMGA1	-1.86
PRDX2	-1.62	EIF3H	-2.34	TSC22D1	2.48	HMGB1	-1.66
PRX	-1.62	EIF4A2	-1.88	TSSC4	1.79	HOMER1	-1.65
RAB31	-1.54	EMC8	-1.70	UCP1	-2.39	HSD17B10	1.93
RAB5A	-1.54	ENPEP	-1.53	UGGT1	-3.16	HSDL2	-1.76
RAB7A	-1.66	EPHX1	-1.53	Uncharact erized protein KIAA1143 homolog	2.01	HSPB3	-1.65
RALB	-2.02	ERLIN2	-1.73	UPF0364 protein C6ORF21 1 homolog	-1.59	IDH2	1.53
RBM10	-1.83	ESD	-1.54	UPF0723 protein C11ORF8 3 homolog	-1.74	IDH3B	1.63
SCRN1	-1.63	ETFA	-1.52	VAMP2	1.52	IKBKB	2.89
SERPINB1A	-1.95	FAS	1.81	VDAC2	-1.59	IL1RAP	-1.94
SERPINH1	-1.72	FGG	-1.61	VDAC3	-1.53	JPH2	-1.53
SLC25A16	-1.82	FLNC	-1.61	WDR1	-1.92	KNG1	-1.53
SLC4A1	-1.54	FNBP1L	1.62	VPS29	-1.62	KRAS	1.69
SMC3	-1.51	GADD45GIP1	1.61	ZC3H18	1.53	KRT19	1.62
STAMBP	-1.68	GARS	-1.61			LDHA	2.04
STRN3	-1.73	GDI1	-1.50			LRRFIP2	-1.61
TF	-1.68	GK	-2.63			LXN	1.53
TUBA1C	-1.73	GLUD1	-2.06			LYPLA1	1.64
TUBB5	-1.75	GNAQ	-1.84			MAP6	-1.62
TWF1	-1.81	GNB1	-2.26			MAPK1	1.59
UPF0235 PROTEIN C15ORF40 HOMOLOG	-1.81	GNB2	-3.28			MARCKS	-1.70

WDR1	-1.55	GNB2L1	-2.31	MCPT1	1.72
XPNPEP1	-2.23	GPD2	-2.38	MDH1	1.68
		GPS1	-2.05	MDH2	1.82
		GPX3	-1.51	MINPP1	-1.50
		GRPCB	4.58	MTCO2	1.60
		H3F3B	-1.69	MTDH	-1.75
		HAGH	-1.62	MUSTN1	-2.15
		HDHD2	1.65	NAMPT	1.68
		HK1	-2.39	NAP1L4	-1.62
		HK2	-2.32	NCL	-1.87
		HMOX1	1.98	NDUFA10	1.62
		HN1	1.60	NDUFAF4	-1.70
		HPCA	1.52	NDUFS1	1.75
		HSPA1A	1.51	NDUFS2	1.83
		HSPA2	1.52	NEXN	-1.62
		HSPA4	-1.58	NOLC1	-2.06
		HSPB6	1.60	NRADD	-1.54
		HYAL1	1.53	NUDT5	1.59
		IDH3G	-2.00	NUP54	1.94
		IFI30	1.50	OPA1	1.57
		IFITM3	1.75	PARVA	1.66
		IG LAMBDA-2 CHAIN C REGION OS	-1.77	PBXIP1	-1.55
		IGFBP5	-1.80	PFN1	1.68
		IGH-1A	-1.87	PPP1R1A	-1.65
		IL17F	1.53	PRPSAP2	2.21
		IMPACT	1.66	PRRC1	1.53
		INSRR	-1.65	PSMA2	1.82
		ISYNA1	-1.93	PSMA4	2.50
		KLKB1	-1.78	PSMA7	1.57
		KRT10	1.91	PSMB4	2.11
		KRT2	2.00	PSMC2	1.78
		LAMB2	-1.52	PSMD11	1.59
		LCMT1	-1.51	PVALB	-1.60
		LGALS3	-2.06	PYURF	-3.17
		LRPPRC	-1.83	QDPR	1.83
		LRRC59	-2.02	RAB11B	2.17
		LTA4H	-2.17	RAB18	1.60
		MACF1	1.51	RAC1	2.15

MAJOR URINARY PROTEIN	-1.80	RBM20	-1.52
MANF	1.55	RPL11	1.55
MAOA	-3.11	RPL18	-1.62
MARCKSL1	1.51	RPL22	-1.73
MATR3	-1.54	RPL38	-1.79
MBNL2	-1.70	RT1-BB	1.52
ME1	-1.87	SAE1	-1.86
MINK1	1.89	SCGB1A1	1.83
MTHFD1	-2.37	SCN1B	-1.91
MYH11	-1.98	SEC22B	1.61
MYH3	-2.29	SEPT8	1.53
MYH4	-1.77	SERPINA6	-1.57
MYH6	-2.44	SIDT2	-1.70
MYH7	-2.11	SLC3A2	1.62
MYH8	-2.05	SMPX	-1.64
MYH9	-1.85	SOD2	1.70
MYL9	1.63	SORBS2	1.64
NAP1L1	1.78	SPTBN2	2.22
NAT1	-1.51	SRPRB	2.19
NCAM1	-1.65	SVIP	-1.50
NDUFA9	-2.16	TMED10	1.98
NHP2L1	1.86	TMED2	1.77
NUP98	-1.71	TNNI1	-1.51
OCLN	1.63	TNNI2	-1.55
PC	-2.09	TOR1AIP1	-1.98
PCSK1N	-1.96	TPM2	-1.53
PCSK2	-2.69	TUBA4A	1.56
PDLIM2	1.51	TUBA8	1.97
PDS5A	1.74	UBA1	1.66
PFKM	-2.51	UPF0729 protein C18orf32 homolog	-1.80
PLD3	-1.94	UQCRC1	1.66
PLIN1	-1.55	USMG5	-1.52
PLP2	1.99	WASL	-1.70
PON1	-2.21	VAT1	1.98
PPIF	-1.59	VCP	1.54
PPP1R1B	1.76	YBX1	-2.32
PPP5C	-1.63	ZC3H15	-1.68

S Table 4 ***Unique regulated protein sin plasma for each test group with a fold change value above 1.5 or under -1.5 for young rats killed three, six and nine month after ^{131}I administration respectively.***

Protein plasma young rats							
**Y3**		**Y6**		**Y6 cont.**		**Y9**	
**Protein**	**Fold change**	**Protein**	**Fold change**	**Protein**	**Fold change**	**Protein**	**Fold change**
ADK	-4.92	A2M	-17.63	KRTDAP	-3.46	Urinary protein 1	-3.33
ANXA1	-1.58	Apolipoprotein A-II	-1.95	LALBA	-1.53	ACP1	-2.29
ANXA2	-2.40	Fibronectin	-1.63	LDHB	-1.56	AKR7A2	-1.67
ARG1	-2.24	ACTA2	-2.73	LIPC	-1.78	ALDOB	-1.55
ARPC2	-1.61	ACTB	-2.70	LTBP1	-1.83	AOX1	-1.99
ATP6V0C	-2.22	ACTN1	-1.64	MAP2K5	-1.50	ATIC	-1.85
CNTN2	-1.59	ACTR3	-2.08	MASP1	-2.70	ATP2A3	-2.08
CRIP1	-1.53	AK1	-2.03	MB	-2.13	BID	-1.83
CSAD	-2.45	ALDOA	-1.57	MMP2	-1.53	BPIFA2	-1.55
CSPG4	-4.01	ANXA5	-1.68	MUG2	-1.99	CALD1	-1.92
CTH	-3.43	APOA1	-2.48	MYOC	-2.16	CAND1	-1.51
CYB5A	-1.61	APOA2	-5.00	NOVA1	-2.18	CANX	-1.87
DBI	-1.55	APOC1	-2.18	P4HB	-1.61	CARHSP1	-1.75
DDT	-1.79	APOC4	-2.12	PARK7	-1.55	CHGA	-1.57
DIEXF	-1.65	APOD	-1.69	PCSK9	-2.75	CHI3L1	-2.18
ENPEP	-1.77	APOE	-1.60	PCYOX1	-1.84	COPS4	-3.29
EZR	-1.64	APOM	-2.62	PGAM2	-2.40	CPQ	-1.65
FAH	-5.08	ARHGDIA	-1.54	PGD	-1.63	CPS1	-2.79
GBP2	-1.55	BASP1	-3.29	PGM1	-1.97	CTSL	-1.60
GSTM1	-1.67	C4BPA	-3.99	PNP	-2.21	CYP27B1	-1.57
GSTM2	-1.71	C4BPB	-4.41	PPIA	-1.77	DLGAP4	-2.01
GSTT2	-1.66	CA3	-1.74	PRKACA	-1.94	EIF1A	-1.73
IL1R2	-2.85	CAP1	-1.95	PROS1	-1.89	ESD	-1.55
IL1RAP	-1.77	CAPN2	-1.91	PSAP	-1.78	ESYT1	-1.60
KDR	-2.16	CAPZA1	-2.09	PSBPC2	-17.23	FAM129A	-2.36
LAMP2	-2.02	CAPZA2	-1.91	PSMA5	-2.28	FASN	-1.55
LGALS1	-3.00	CCT4	-2.05	PSMA7	-1.62	FTH1	-2.53
LGMN	-1.50	CDH1	-1.59	PSMB8	-1.66	G6B	-2.92
MSLN	-1.66	CDH17	-1.52	PTPN11	-2.28	GLO1	-2.00
Protein-glutamine gamma-glutamyltransferase K	-1.61	CKB	-1.79	PYGM	-2.42	GP1BB	-1.56
NF1	-1.62	CLIC1	-1.53	RAB11A	-2.00	GP5	-2.29
ORM1	-2.33	CLTC	-1.76	RAB27B	-1.70	GPX1	-1.93

OSMR	-3.59	CMPK1	-1.70	RALB	-2.51	H1F0	-1.94
PLCG2	-1.83	CNN3	-1.51	RNH1	-1.78	HPSE	-1.68
PON3	-2.76	CORO1A	-2.82	RPL3	-1.71	IMPA1	-1.61
PPP1R7	-1.64	CORO1B	-2.22	S100A6	-1.62	LGALS5	-4.31
PROCR	-1.80	CP	-1.57	SERPINA 11	-1.65	LMNB1	-1.64
RASA3	-1.79	CRK	-1.64	SLC3A2	-2.22	MDH2	-1.51
RLC-A	-1.62	CTSC	-1.60	SRC	-2.37	MYH9	-1.53
SDPR	-1.52	DBNL	-1.75	TGFB1	-2.07	NIT1	-1.51
SELL	-1.77	DNAH12	-1.51	THBS4	-1.58	PACSIN2	-1.85
SERPINA1	-1.53	DPEP2	-3.35	TMOD2	-2.06	PLCB4	-1.96
SLC25A5	-1.87	DPP3	-2.39	TPI1	-1.66	PPIF	-1.53
SPRR1A	-1.61	EFEMP1	-2.17	TPM3	-2.04	PPP1R12A	-1.77
SPTBN2	-1.69	ENO3	-2.87	TTR	-1.79	PPP2R1B	-1.53
TES	-2.22	F12	-2.81	TUBA1C	-1.90	PRDX2	-1.97
TGM1	-1.51	F2	-2.08	TUBA4A	-1.60	PRDX5	-1.60
T-kininogen 2	-1.64	FABP4	-1.55	TUBB2A	-2.02	PRKCA	-1.85
VWF	-1.97	FCN1	-1.58	TXNL1	-1.51	PSMA3	-1.62
XK	-9.68	FDPS	-1.74	UBA1	-1.52	PSMB3	-1.56
		FKBP4	-1.58	UBE2V2	-1.75	PSMB7	-1.63
		FN1	-4.87	VCL	-1.87	RAN	-1.59
		GAPDH	-1.90	XPNPEP 1	-1.57	RAP2B	-2.00
		GMPR	-1.68	YWHAB	-2.41	RHD	-1.55
		GSTM5	-1.68	YWHAG	-1.80	RPL6	-2.00
		GYG1	-1.71	YWHAH	-1.86	RRAS	-1.69
		HABP2	-2.31	YWHAZ	-1.63	S100A8	-1.73
		HINT1	-4.14			SELP	-1.73
		HIST1H1E	-2.11			SHBG	-1.52
		HIST1H2BA	-1.93			SLC4A1	-1.56
		HIST1H4B	-1.75			SMPX	-1.62
		HP	-4.03			SPP1	-2.02
		HSPA4	-1.61			SPP2	-1.56
		IGFBP3	-1.61			TAOK3	-1.78
		IGFBP5	-1.52			TMSB4X	-1.73
		INHBC	-1.84			TPP2	-2.15

S Table 5 ***Unique regulated thyroid transcripts for each test group with a fold change value above 1.5 or under -1.5 for adult rats killed three, six and nine month after ^{131}I administration respectively.***

Thyroid transcripts adult rats											
A3		A6		A9		A9 cont.		A9 cont.		A9 cont.	
Gene	Fold change	Gene	Fold change	Gene	Fold change	Gene	Fold change	Gene	Fold change	Gene	Fold change
Col17a1	3.80	*Bmp8a*	1.59	*Abcf2*	1.54	*Fbxo40*	3.18	*Lrrc14b*	1.95	*Relt*	2.22
Dmkn	4.48	*Bpifa1*	2.16	*Acadm*	1.33	*Fgf5*	-1.61	*Lrrc14b*	2.87	*RGD1307461*	1.54
Hao2	-4.21	*C4bpa*	-1.30	*Ache*	2.62	*Fhl3*	2.04	*Lrrc30*	3.04	*RGD1310495*	2.48
Kap	-5.15	*Fhl2*	1.16	*Acsl6*	2.26	*Fhod3*	2.02	*Lrrc3b*	1.51	*RGD1310507*	1.20
Lipf	-3.94	*Gh1*	-1.67	*Adamts17*	-2.23	*Filip1*	1.82	*Lrrn4*	1.97	*RGD1311874*	1.83
LOC102556288	2.75	*Gk*	-1.67	*Adamts20*	2.08	*Fndc5*	2.62	*Lsmem1*	2.87	*RGD1560334*	1.95
LOC360228	-3.38	*Klk1c2*	-2.12	*Adtrp*	1.80	*Foxo6*	1.51	*Lynx1*	2.16	*RGD1561689*	-2.14
Miox	-5.30	*Klk1c4*	-1.68	*Afap1l1*	1.85	*Fsd2*	3.26	*Lysmd4*	2.23	*RGD1561777*	1.69
Mup4	2.15	*Krt13*	3.67	*Afap1l2*	-1.93	*Gfpt2*	1.30	*Mafa*	2.54	*RGD1561778*	1.61
Pax4	2.36	*Lgals7*	3.06	*Agl*	1.64	*Ggct*	-1.54	*Mamstr*	1.89	*RGD1564541*	-1.39
Sbsn	3.43	*LOC100360165*	2.42	*Akr1c3*	2.22	*Ggt6*	2.49	*Map2k6*	1.32	*RGD1564899*	2.76
Slc7a13	-3.26	*LOC171161*	1.70	*Aldh1a7*	4.10	*Gmcl1l*	2.29	*Map3k10*	1.69	*RGD1564899*	2.78
Slpi	3.63	*Npw*	1.11	*Alms1*	-1.48	*Got1*	1.93	*Marveld2*	-1.43	*RGD1565323*	2.89
Sprr1a	3.32	*RGD1562234*	3.32	*Alox12e*	-2.19	*Gpcpd1*	2.11	*Masp1*	1.77	*RGD1584023*	-1.97
Tg	2.45	*Scgb3a1*	1.21	*Alpk3*	1.86	*Gpr88*	1.75	*Mc5r*	2.85	*Rgma*	1.44
Umod	-3.61	*Slc20a1*	-1.05	*Amy1a*	2.06	*Gpt2*	2.04	*Mettl21c*	2.63	*Rprd1b*	-1.71
		Upk1b	1.36	*Angpt2*	1.84	*Grid1*	-1.48	*Mex3b*	-1.64	*Ryr1*	2.69
		Wfdc10	1.43	*Ankle2*	-1.18	*Gstm5*	1.10	*Mfsd10*	-2.13	*Ryr2*	2.62
				Ankrd1	2.46	*Gucy2g*	2.70	*Mgat5*	-1.34	*S100b*	2.30
				Anxa10	2.93	*Gucy2g*	2.84	*MGC109340*	1.35	*Sacs*	2.01
				Aqp4	1.81	*Gypc*	1.95	*MGC116197*	1.91	*Samd5*	-1.38

Aqp4	2.02	*Gys1*	2.15	*Milr1*	2.10	*Scn1a*	1.56
Aqp4	2.86	*Gzmc*	1.92	*Mlip*	2.19	*Scn1b*	2.71
Arc	1.67	*Habp4*	1.96	*Mrm1*	-1.46	*Scn4b*	2.82
Arhgap26	-1.65	*Hhatl*	2.42	*Mroh4*	-2.21	*Scn8a*	-1.62
Arpp21	2.21	*Higd1a*	1.63	*Ms4a12*	-2.83	*Sdk2*	-1.54
Arpp21	2.23	*Hpgds*	1.83	*Mss51*	2.60	*Sec1*	1.89
Arpp21	2.33	*Hrasls*	2.33	*Mthfd2*	-1.72	*Sec31b*	2.41
Arrdc2	1.55	*Hsf4*	1.64	*Mthfr*	-1.42	*Sema5a*	-1.30
Art3	2.24	*Hspb8*	1.75	*Myadml2*	2.23	*Sema7a*	1.53
Art5	1.87	*Ifitm10*	1.36	*Myh2*	3.44	*Serpinb3*	2.16
Arx	2.80	*Ifna2*	2.59	*Myh6*	5.86	*Sgca*	1.83
Asb10	2.19	*Il1r1*	-1.44	*Myh7*	5.50	*Sh3bgr*	1.88
Asb14	3.12	*Ip6k3*	2.04	*Myl2*	5.22	*Sh3rf2*	3.37
Asb16	2.61	*Ipcef1*	-1.53	*Mylk2*	2.66	*Shisa3*	1.58
Atl2	1.42	*Iqsec3*	-1.33	*Mylk2*	3.31	*Sim2*	-2.57
Atp1b2	2.99	*Irf8*	-1.58	*Myo18b*	2.56	*Six1*	1.75
Atp1b4	1.88	*Irs4*	-1.91	*Nefh*	-1.60	*Ski*	-1.44
Auh	1.59	*Irx5*	2.43	*Nepn*	2.61	*Slc25a42*	1.72
Aven	1.27	*Izumo1*	2.09	*Neu2*	3.29	*Slc38a4*	1.69
Bap1	1.40	*Jph1*	2.15	*Nexn*	2.09	*Slc41a3*	2.21
Best3	1.85	*Jph2*	3.00	*Nfil3*	1.57	*Slc43a1*	1.54
Bmpr1b	-1.36	*Kb23*	-2.11	*Nkain1*	1.88	*Slc8a3*	2.26
Btbd17	-2.22	*Kbtbd12*	2.14	*Nkx2-5*	2.29	*Slc9a2*	1.88
Bves	2.48	*Kcna7*	2.19	*Nr1d1*	1.82	*Slco4c1*	-1.62
Cacna2d1	2.54	*Kcnb2*	-1.57	*Nrep*	2.09	*Smarca1*	-1.57
Cacng1	2.68	*Kcnc4*	2.17	*Nrsn1*	-1.78	*Smpdl3b*	3.02
Cacng6	2.45	*Kcnj11*	2.17	*Ntn1*	-1.50	*Smtnl2*	2.84
Cacng6	2.64	*Kcnj12*	2.39	*Olr1091*	2.53	*Snta1*	1.55
Cadps	-1.49	*Kcnk2*	2.44	*Olr184*	-2.20	*Sorcs1*	-1.18
Calu	-1.37	*Kcnma1*	1.92	*Opn4*	1.51	*Sox6*	1.97

Capn3	1.94	*Kcnq3*	-1.65	*Ostn*	2.07	*Spata2*	1.55
Ccl1	2.88	*Kl*	-2.26	*Otud5*	-1.24	*Speg*	2.53
Ccna1	1.64	*Klhl21*	1.85	*P2ry1*	2.18	*Spetex-2F*	1.88
Ccr1	1.69	*Klhl30*	2.39	*Palld*	2.70	*Srcrb4d*	1.32
Cd55	1.56	*Klhl30*	3.53	*Pax1*	-2.19	*St8sia5*	2.60
Cd55	2.47	*Klhl31*	2.01	*Pcyox1*	1.20	*Stox2*	-1.51
Cdh15	2.28	*Klhl38*	2.41	*Pcyox1l*	-1.39	*Stradb*	1.74
Cdkn2b	2.20	*Klhl40*	2.21	*Pdcd6ip*	-1.41	*Sugt1*	-1.21
Cers1	2.73	*Klrc3*	2.32	*Pde4dip*	2.94	*Svil*	1.63
Cfl2	1.93	*Ksr1*	1.55	*Pdlim7*	2.34	*Sypl2*	2.83
Chmp4b	-1.38	*Ky*	2.07	*Pdzd8*	-1.34	*Taok3*	-1.54
Chrnd	2.24	*Lbx1*	3.50	*Pfkfb1*	1.91	*Tceal5*	1.75
Chst11	-2.21	*Lhx4*	2.11	*Pgm1*	2.53	*Tceal7*	1.95
Clcn1	3.07	*Lilrb3l*	2.01	*Phf8*	-1.62	*Tdrd1*	2.09
Clcn4	1.70	*Lmod1*	1.83	*Phka1*	2.69	*Tmem182*	1.57
Cnrip1	1.22	*Lmod1*	2.18	*Phospho1*	1.91	*Tmem196*	2.44
Cpa4	1.51	*Lnx1*	1.79	*Phtf2*	1.52	*Tmem38a*	2.26
Cplx2	-1.74	*LOC100360457*	-1.52	*Phyh*	1.81	*Tmprss3*	-1.89
Cpt1b	2.14	*LOC100909944*	1.92	*Pik3cd*	1.99	*Tnfrsf22*	1.33
Crhr2	2.04	*LOC100910823*	-1.72	*Pira2*	2.14	*Tnnc1*	3.50
Crtac1	-1.44	*LOC100911353*	2.90	*Pitx2*	2.40	*Tnnt1*	3.96
Ctnnd1	-1.71	*LOC100911353*	3.28	*Pkdcc*	1.68	*Tnnt1*	3.98
Cyp2d1	2.25	*LOC100911864*	2.76	*Pkm*	2.30	*Tnr*	-1.35
Cyp2d5	2.64	*LOC102547963*	-2.37	*Plbd1*	2.08	*Top1*	-1.54
Dach1	-1.15	*LOC102550394*	-1.39	*Plek*	1.85	*Tp53inp2*	1.70
Dcun1d2	2.06	*LOC102551314*	1.71	*Plekhf2*	1.90	*Trem3*	1.79
Dcun1d2	2.15	*LOC102554096*	2.15	*Plin5*	2.23	*Trib1*	-1.22
Ddah1	1.82	*LOC102555594*	2.51	*Plk2*	-1.39	*Trim16*	1.80
Ddr1	-1.50	*LOC102555814*	2.15	*Pnmal2*	-1.82	*Trim63*	2.51
Dennd2c	1.97	*LOC290595*	1.91	*Pnpla3*	1.31	*Trim7*	1.99

Diaph1	-1.28	*LOC298139*	2.15	*Popdc3*	2.38	*Tspyl2*	-1.54
Dmbt1	1.20	*LOC298139*	2.15	*Ppp1cb*	1.58	*Tspyl5*	-1.53
Dnajb5	1.99	*LOC307263*	2.29	*Ppp1r3a*	2.16	*Ttyh2*	1.95
Dnajc30	1.22	*LOC367117*	1.14	*Ppp2r3a*	1.90	*Ubac1*	1.83
Dupd1	2.18	*LOC367381*	1.76	*Prkacb*	-1.50	*Ube2d1*	1.60
Dusp10	1.69	*LOC367381*	2.05	*Prkag3*	2.58	*Ube2ql1*	1.89
Dusp27	2.14	*LOC500354*	1.99	*Prkag3*	3.08	*Unc13a*	-1.76
Ece1	-1.74	*LOC500959*	1.89	*Prob1*	1.45	*Ush1c*	2.11
Egln3	2.26	*LOC501223*	2.97	*Pth*	-3.54	*Usp46*	-1.43
Ehbp1l1	1.72	*LOC501224*	1.60	*Ptpdc1*	-1.23	*Usp6nl*	-1.63
Eif4e3	2.02	*LOC501396*	2.43	*Ptprf*	-1.41	*Wasf3*	-1.61
Elmsan1	1.63	*LOC56764*	1.88	*Rab28*	2.15	*Vgll2*	2.85
Emx2	1.60	*LOC680273*	-2.36	*Rangrf*	1.45	*Wif1*	1.76
En1	3.24	*LOC683753*	1.30	*Rasgrp3*	1.88	*Vldlr*	2.05
Epdr1	1.82	*LOC685580*	1.87	*Rbm15b*	-2.44	*Vtcn1*	2.53
Epm2a	2.00	*LOC687797*	2.44	*Rbm20*	3.31	*Wwox*	-1.42
Erc2	-1.75	*LOC688869*	1.22	*Rbm24*	2.41	*Xkr4*	1.57
Fabp3	2.35	*LOC689459*	1.87	*Rbm24*	2.60	*Yipf7*	2.72
Fads6	1.83	*LOC689519*	1.69	*Rcan2*	1.39	*Zbtb47*	2.00
Fam198a	2.20	*LOC691414*	1.58	*Rcan3*	-1.58	*Zik1*	-1.46
Fam220a	1.76	*LOC691414*	2.34	*Rcor2*	1.76	*Zpld1*	-2.70
Fam65c	-2.24	*Lpar3*	1.68	*Rcsd1*	2.11		

S Table 6 ***Unique regulated thyroid proteins for each test group with a fold change value above 1.5 or under -1.5 for adult rats killed three, six and nine month after ^{131}I administration respectively.***

Thyroid protein adult rats							
A3		A3 cont.		A4		A9	
Protein	Fold change	Protein	Fold change	Protein	Fold change	Protein	Fold change
A1I3	-2.54	LYRM9	-1.79	ACP1	-1.82	AK3	-1.53
ABHD14B	-2.23	MACF1	1.53	ACTA1	1.76	ATP5H	1.77
ACAA2	-1.87	MANF	1.80	ACTB	1.63	BCAM	1.91
ACADSB	-2.23	MAP1A	1.84	ACTN4	1.96	BGN	1.97
ACAN	2.33	MAP2	2.35	AGA	-2.22	CALD1	4.49
ACAT1	-2.22	MAP2K5	-1.99	AHSG	-1.70	CMBL	1.80
ACLY	-2.70	MAP4	1.69	AIP	1.99	COQ9	-11.2
ACO1	-3.59	MAP6	1.84	ANP32A	-2.12	COX5A	1.68
ACOT2	-3.41	MCPT1	-2.00	ANXA1	1.65	CRELD1	3.11
ACSF2	-1.83	ME1	-2.42	ANXA4	1.65	CYB5A	1.58
ACSL1	-3.33	MED22	2.80	ANXA5	-1.54	CYCS	-1.71
ACTR1A	-1.55	MGLL	-1.93	APOE	-2.66	DLST	1.65
ACTR2	-2.27	MINK1	1.90	ASRGL1	-1.59	DSTN	-2.01
ACY3	-2.75	MLEC	-3.57	ATP5E	1.74	DYNC1LI2	1.57
ADAM10	-1.51	MTDH	1.62	BNIP1	1.53	EIF5A	1.85
ADH5	-4.83	MTHFD1	-1.55	BSG	-1.97	FKBP1A	1.95
AGFG1	1.78	MTPN	1.50	CANX	-1.80	FTL1	1.53
AHCY	-4.11	MYBPH	-1.70	CAPG	1.75	GPD1	1.69
AIF1	1.70	MYH4	-1.55	CAPZB	1.64	GSTA5	-1.94
AIFM1	-3.06	MYL1	1.67	CCDC91	1.53	H1F0	-1.55
AK8	-1.71	MYL9	1.55	CCT4	2.01	HINT1	-1.78
AKR1C9	-2.00	NAP1L1	2.34	CD59	-2.73	HSP90AA1	1.55
ALCAM	1.53	NDUFA9	-2.77	CFI	-1.96	HSPB7	1.97
ALDH1A1	-2.66	NES	1.92	CHGA	-1.89	LGALS3BP	1.77
ALDH3A2	-2.45	NEXN	1.55	CKMT2	2.20	LMNB1	1.86
ALDH5A1	-2.53	NIT2	-1.57	CLIC4	-1.82	MTCO2	-1.61
ALDH7A1	-1.68	NKX2-5	1.55	CLIC5	-1.73	MYH7	1.97
AMPD1	-3.28	NMT1	2.26	CNN3	-1.69	MYLK2	1.55
AMPH	-1.61	NOL3	1.70	COQ7	-1.56	MYO9B	1.68
ANP32B	1.58	NONO	-2.08	CPE	1.61	NDUFV2	1.80
ANP32E	1.59	NPTN	1.74	CRIP2	-1.57	PRELP	-2.36
ANXA8	-1.56	NRGN	1.79	CRKL	1.85	PRKCDBP	-1.58
AOC3	-1.70	NUCB2	1.54	CST3	-2.28	PRPH	1.78
AP1B1	-1.94	NUPL1	-1.65	CTTN	1.86	PRX	3.03
AP2A2	-2.15	OBP1F	1.91	DCTN2	-2.61	PSME2	3.22
AP2M1	1.56	OCLN	1.80	DDAH2	-1.68	PURA	1.57

APCS	-2.76	OCM	7.11	DNM1L	2.36	RPS28	1.90
APLP2	2.53	OPA1	-1.51	DPP7	2.19	SERPINA3N	1.67
APMAP	-3.10	OPLAH	1.86	EMC10	-1.59	ST13	1.84
APOA1	-2.27	P33MONOX	1.52	EMC2	1.86	SUB1	-1.54
APP	1.54	PA2G4	-2.03	ENPP3	1.52	TMOD1	2.47
AQP1	-1.77	PABPC1	-1.87	ERP29	-1.70	TRIM72	1.72
ARF3	-2.69	PABPN1L	-1.85	FTH1	-1.81	UBE2V2	1.70
ARF5	-1.87	PAF1	1.60	FUBP1	1.71		
ARFGAP1	1.60	PC	-1.80	GGT5	-2.22		
ARFGAP2	1.77	PCCA	-3.20	GLO1	-2.64		
ARFIP2	-1.54	PCCB	-4.41	GOSR2	1.67		
ARPC1B	-1.58	PCP4	1.96	GPX1	1.70		
ARPC2	-1.95	PCSK2	-1.68	GSTZ1	1.59		
ARPP19	2.15	PDHB	-3.01	H2AFZ	-2.26		
ASL	-2.25	PDK2	-1.81	HAGH	-1.50		
ATIC	-1.90	PDP1	-1.90	HIBADH	2.01		
ATP1A2	-1.68	PDS5A	2.12	HIST1H1E	-2.58		
ATP1B2	1.51	PDXK	-2.59	HMMR	3.23		
ATP5G1	-2.02	PEPD	-2.89	HNRNPF	-1.85		
ATP5J	1.99	PFKM	-4.18	HNRNPM	2.49		
ATPIF1	1.83	PGD	-1.93	HSPA9	1.73		
BAG1	1.66	PGM1	-1.51	HSPE1	-1.75		
BAIAP2	1.62	PHGDH	-1.58	IAH1	-1.72		
BANF1	1.60	PHKA1	-1.74	Ig kappa chain c regio. A allele	1.63		
BCAS1	1.81	PKIA	1.58	IL1RAP	-1.96		
BCAT2	-1.99	PKM	-2.75	IMMT	1.85		
BCKDHA	-3.00	PLBD2	1.50	KHSRP	1.53		
BCKDHB	-2.01	PLD3	-1.85	KNG1	-1.90		
BID	1.72	PLET1	1.89	KRT10	-3.00		
BPNT1	-1.90	PLG	-1.56	LAMP1	-1.76		
CA5B	-2.43	PMPCA	-2.41	LASP1	-1.51		
CAP1	-1.61	PON1	-2.83	LMAN1	-2.50		
CAPN1	-1.85	PPFIA3	1.51	LRPAP1	-1.61		
CAST	1.86	PPP1R11	1.82	LRRC14	-1.83		
CAV1	-1.56	PPP1R1A	1.62	LYPLA1	1.77		
CCBL2	-3.53	PPP1R1B	2.70	LZIC	-2.54		
CCT5	-1.72	PPP1R2	1.68	MAP1	-1.81		
CD36	-1.56	PPP3R1	1.65	MAPK3	7.10		
CD44	1.87	PPP5C	-1.58	MAPT	-2.18		
CD47	-1.72	PRDX2	-1.83	MDH2	2.11		
CD63	1.89	PRDX3	-1.95	MIF	-1.55		
CD99L2	1.79	PRKACA	-2.59	MYH9	2.01		
CDC42EP1	-1.53	PRKAG1	-3.15	NAMPT	1.84		
CDNF	1.52	PRKAR1A	-1.81	NAP1L4	-2.01		
CDV3	1.80	PRPS1	-1.55	NDUFA10	-1.91		

CELF1	-1.83	PSAP	1.68	NDUFS1	2.14
CES1C	-1.95	PSBPC2	2.92	NDUFS2	3.16
CHCHD4	2.12	PSMA2	-1.68	NDUFS4	-1.85
CHGB	1.78	PSMA4	-1.72	NEFM	-2.37
CLDN3	2.08	PSMA6	-1.96	NPM1	-2.07
CLNS1A	1.84	PSMB3	-1.83	PDAP1	-1.81
CLTA	1.52	PSMB6	-2.24	PDLIM7	-1.61
CLTB	1.57	PSMD1	-1.84	PEBP1	-1.73
CLYBL	-1.62	PTBP1	-2.03	PFN1	1.65
CMA1	-2.33	PTMS	2.62	PGRMC1	-1.94
CNP	-2.73	PTPN11	-2.14	PIR	4.58
COL1A2	-1.77	PYGB	-2.98	PLS3	1.53
COQ6	-1.91	RAB14	-2.32	PLVAP	2.21
CORO1B	-3.24	RAB1A	-2.16	PODXL	-2.21
CORO6	-1.54	RAB28	-1.65	PPM1B	1.78
CP	-2.32	RAB2A	-3.40	PPP1CA	2.01
CPA3	-1.72	RAB5A	-1.69	PPT1	1.65
CPT2	-2.85	RAB6A	-1.91	PRDX3	2.12
CRYAB	1.51	RAC1	-2.24	PRDX6	2.04
CRYL1	-1.99	RALB	-2.31	PRRC1	2.00
CSAD	-2.09	RAN	-3.17	PSMA3	1.70
CSNK2A1	-2.19	RAP2B	-2.08	PSMA5	1.61
CTBP1	-2.10	RARS	-2.03	PSMC5	1.90
CTSD	-2.15	RBBP7	-1.75	RAD23B	-1.81
CUL5	-1.66	RGS9	2.54	RALA	7.17
CYB5B	1.51	RMDN1	-1.53	REXO2	1.55
CYB5R1	-2.14	RPL10	-1.99	RPS10	2.63
DCPS	-1.64	RPL10A	-2.02	RPS19	-1.51
DCXR	-2.12	RPL11	-1.68	RPS23	1.73
DDOST	-1.70	RPL12	-1.51	RPS25	-1.88
DECR1	-4.25	RPL13	-1.74	RT1-AW2	1.88
DES	1.54	RPL14	1.74	S100A6	-1.76
DLGAP4	1.55	RPL17	-2.63	SCG3	-3.02
DNAAF2	-2.55	RPL18	-1.56	SCN4B	-3.14
DNAH12	-3.96	RPL18A	-1.54	SEC22B	1.86
DNAJA1	-1.97	RPL3	-4.58	SERPINA6	-1.55
DOHH	1.93	RPL35A	-1.63	SKP1	-1.62
DPP3	-1.60	RPL38	-1.65	SLC3A2	2.08
DPP4	-2.50	RPL9	-2.88	SNCG	-2.09
DR1	1.74	RPN2	-3.23	SOD2	-1.66
DYNLRB1	1.61	RPS14	-1.57	SYNCRIP	2.06
ECH1	-1.72	RPS18	1.52	TCEB1	-1.58
EEF1A1	-4.27	RPS2	-2.95	TCEB2	-1.56
EEF1G	-1.84	RPS3	-2.28	TFAM	-5.46
EEF2	-2.36	RPS4X	-2.38	THRSP	-2.27
EFHD2	1.61	RPS8	-2.67	TNNI2	-2.01

EHD2	-1.58	RRAS	-1.51	TNNT3	-1.84
EIF3B	-2.57	RT1-BB	-1.57	TOMM34	1.92
EIF3E	-1.54	RTN4	-2.56	TPM3	-1.65
EIF3H	-2.21	RWDD1	1.60	TPM4	-1.65
EIF4EBP1	1.62	RWDD4	1.57	TTR	-1.73
EMB	2.04	S100A11	2.18	TUBB4B	3.27
EMC8	-1.83	S100A4	1.76	UBAC1	-1.64
ERLIN2	-2.64	S100A8	1.71	UBE2N	-1.88
ES1 protein homolog. mitochondrial	-1.68	S100A9	1.50	UBQLN1	1.72
ESD	-2.63	SBDS	-2.51	UCHL1	-2.52
EXOC7	-1.72	SCGN	-1.50	UFC1	1.85
EZR	-1.53	SDC2	1.88	UQCRFS1	-1.63
FAHD1	-1.54	SDC4	1.67	USO1	2.25
FAHD2	-1.69	SDHA	-4.35	VAMP8	-1.59
FAM195B	1.89	SEC13	-2.55		
FAM213A	-2.37	SEC31A	-1.50		
FAM98A	-1.68	SEPT2	-2.47		
FAS	1.77	SEPT7	-1.89		
FCER1G	1.70	SEPT11	-1.74		
FGFR1OP	1.57	SEPT15	1.90		
FH	-1.62	SERPINA10	-1.57		
FHL1	-1.56	SERPIND1	-1.76		
FKBP4	-1.77	SFR1	1.52		
FLNC	-1.55	SGCE	-1.78		
FMR1	1.87	SIKE1	1.55		
FN1	-2.25	SIL1	1.62		
Fuctinin-3 (fragment)	1.63	SIRPA	1.71		
FXYD1	1.54	SLC25A1	-2.58		
FXYD2	-2.23	SLC25A11	-3.04		
G4	2.17	SLC25A16	-2.41		
G6PDX	-1.55	SLC25A20	-2.62		
GADD45GIP1	2.03	SLC25A3	-2.91		
GAP43	1.57	SLC25A4	-2.46		
GAPDH	-2.00	SLC25A5	-2.70		
GARS	-2.48	SLK	2.00		
GCLM	-1.80	SMARCE1	1.54		
GDI2	-2.62	SMC3	-1.99		
GGT1	-5.42	SMIM13	1.55		
GK	-1.64	SMPD3	2.21		
GLRX	1.68	SNAP23	1.61		
GLTP	1.68	SPTBN2	-1.50		
GLUD1	-2.58	SQSTM1	1.64		
GNAI2	-1.62	SRA1	1.68		
GNAS	-1.55	SRPRB	-1.67		
GNB2L1	-3.06	SSR1	1.64		

GOLIM4	1.76	SSR4	-1.62
GPD2	-3.70	STBD1	-2.15
GPI	-1.74	STK3	1.77
GPS1	-1.74	STX4	1.57
GRPCB	2.08	SUOX	-2.05
GSS	-3.20	SZRD1	1.77
GSTA1	-7.18	TCEB3	1.70
GSTT2	-1.83	TCP1	-3.64
GTF2A1	1.64	TIMM21	-1.69
HADH	-2.74	TIMM22	-1.75
HADHA	-3.52	TIMM8A	1.62
HADHB	-5.74	TINAGL1	-1.58
HK1	-2.25	TMED5	2.02
HK2	-2.03	TMED7	-1.96
HMGA1	1.53	TMEM109	2.36
HMOX1	1.66	TMEM43	-2.72
HN1	1.97	TMSB4X	1.87
HN1L	1.68	TPT1	1.67
HNRNPA1	-2.59	TSC22D1	1.55
HP	-1.75	TSC22D3	1.63
HS3ST2	-3.08	TTGN1	1.67
HSD17B10	-1.65	TUBA1C	-2.13
HSPA4	-1.50	TUBB5	-1.63
HSPB1	1.62	TWF1	-2.12
HYAL1	1.74	TXN	1.57
IDH3B	-1.58	UBA5	-1.56
IDH3G	-2.21	UBE2F	1.54
IFI30	1.78	UBXN4	1.62
IFITM3	2.21	UCP1	-3.62
Ig lambda-2 chain c region	-1.78	UGGT1	-2.37
IGFALS	-1.55	Uncharacterized protein c12orf43 homolog	1.54
IGG-2A	-2.19	Uncharacterized protein c9orf40 homolog	2.51
IL17F	5.38	Uncharacterized protein kiaa1143 homolog	2.20
IMPACT	2.53	Upf0364 protein c6orf211 homolog	-1.80
INSRR	-1.90	Upf0723 protein c11orf83 homolog	-2.08
IRGC	1.97	Upf0729 protein c18orf32 homolog	1.59
ISYNA1	-1.80	VAT1	-1.73
ITIH3	-1.98	VDAC2	-1.63
KCNIP3	1.96	VDAC3	-1.88
KLHL41	-1.82	WDR1	-3.27
KRT75	1.79	Vomeromodulin (fragment)	1.82

LAMP2	1.58	VPS29	-2.45
LAP3	-1.67	VTI1B	1.66
LDHA	-1.95	XPNPEP1	-1.75
LDHB	-2.26	YBX3	1.65
LIPE	-1.53	YIPF3	2.68
LONP1	-2.48	ZC3H15	1.64
LRRFIP2	1.67	ZC3H18	1.91
LTA4H	-2.59	ZRANB2	1.60
LYPD3	4.95	ZWINT	1.84

S Table 7 ***Unique regulated proteins in plasma for each test group with a fold change value above 1.5 or under -1.5 for adult rats killed three, six and nine month after ^{131}I administration respectively.***

Plasma adult rats							
A3		A6		A6 cont.		A9	
Protein	Fold change	Protein	Fold change	Protein	Fold change	Protein	Fold change
A2M	-23.18	ABCC6	-2.29	PSMB3	-2.12	ACLY	-2.83
AK1	-2.19	ACO2	-1.67	PSMB7	-1.51	ACPP	-2.68
ANXA5	-2.36	ALAD	-1.67	PSMB9	-1.74	ACTR1A	-3.82
APOA1	-2.29	ALDH7A1	-2.75	RNH1	-1.70	AGT	-1.65
APOD	-1.50	ALDH9A1	-1.59	RPL6	-1.57	AHSG	-1.66
APOM	-1.88	APOA5	-2.38	RRAGA	-1.70	ARF5	-2.10
ATRN	-1.60	APOB	-2.47	SEPTIN11	-1.52	ATP5B	-3.65
BIN2	-1.89	APRT	-1.80	SEPTIN2	-1.88	AUNIP	-1.92
BLVRA	-2.42	ASS1	-2.06	SEPTIN7	-2.02	BTD	-1.53
CA3	-1.91	BPIFA2	-2.36	SERPINA7	-1.52	CALD1	-1.71
CALR	-1.74	C1QA	-1.77	SERPIND1	-1.71	CAPZB	-1.69
CAPG	-1.85	C1QB	-1.76	SERPINE2	-2.24	CCT4	-2.01
CAPN2	-1.81	C1S	-1.51	SNED1	-2.46	CD300A	-1.71
COMP	-1.64	C5	-1.88	SPR	-1.62	CES1C	-1.51
CORO1A	-1.90	CA2	-2.39	TFRC	-1.53	CES1D	-1.79
CORO1B	-1.69	CAPN1	-1.73	TG	-2.66	CLIP2	-3.44
CSTB	-1.65	CCL6	-2.84	Alpha-aminoadipic semialdehyde dehydrogenase	-1.58	CMPK1	-4.17
CTSB	-1.53	CEACAM1	-2.04	TSKU	-1.52	CPQ	-1.67
CYCS	-2.09	CELSR3	-1.57	VAT1	-1.51	CRIP1	-1.61
DSG4	-2.15	CHI3L1	-1.60	XYLB	-2.13	CSAD	-1.79
EFEMP1	-2.03	CKMT2	-1.57			DLGAP4	-1.95
EML2	-1.52	CLTA	-1.95			DNAH12	-2.05

FABP3	-1.88	CNDP2	-2.04	DNM2	-3.71
FABP4	-2.73	COL1A1	-1.71	EEF2	-1.52
G6PDX	-2.24	COPS4	-1.62	EIF4A2	-2.20
GAPDH	-1.65	CPB2	-2.15	ESD	-1.60
GMPR	-1.57	CPN1	-1.52	FKBP4	-1.50
HSPA4	-1.55	CRYAB	-2.70	GBP2	-2.67
ILK	-2.19	CSRP1	-2.05	GP5	-1.62
LDHB	-1.69	DHTKD1	-4.30	GSR	-1.77
LGALS1	-2.36	DMBT1	-2.98	GSTM1	-2.04
LUM	-1.63	DPYS	-1.51	GSTT2	-1.58
MB	-2.57	ECM1	-2.92	HAL	-2.46
MDH1	-1.51	EML1	-1.64	HNRNPA2B1	-9.35
MINPP1	-1.70	ENO1	-1.81	HNRNPK	-5.74
MYH10	-1.79	EPHX1	-2.26	HPX	-1.55
PAICS	-1.58	ESYT1	-1.74	HSP90AB1	-2.37
PARK7	-1.93	F12	-2.69	HSPA5	-1.68
PDIA3	-1.52	FABP1	-1.51	HSPA8	-2.08
PDLIM1	-1.95	FCN2	-1.90	HYAL1	-1.55
PEBP1	-1.53	FSTL1	-1.86	IL1RAP	-1.72
PFN1	-2.03	FUCA1	-1.53	KDR	-2.01
PGAM2	-2.93	GLYCAM1	-2.46	KPNB1	-2.13
PGM1	-2.13	GPI	-1.92	LAMP2	-1.66
PKLR	-2.41	GPR116	-1.67	LGMN	-1.74
PLA2G2A	-1.53	HIST1H1B	-1.77	LRRC59	-2.34
PLCG2	-2.21	HMGN2	-1.81	LTA4H	-1.68
PLEK	-1.73	ICAM1	-2.14	LYN	-1.77
PNP	-1.55	IGFBP6	-1.55	LYZ1	-1.56
PRDX2	-1.57	KALRN	-1.85	MAK	-2.10
PRDX6	-1.90	KNG1	-1.95	MSLN	-1.99
PRKACA	-1.69	L1CAM	-1.68	MSN	-2.50
PTPN11	-2.20	LGALS3	-1.61	MYOC	-1.82
PYGM	-2.68	LGALS5	-1.52	P4HB	-2.63
RAB27B	-2.04	Complement c1s subcomponent	-1.69	PGAM1	-4.88
RLC-A	-1.79	LMNB1	-1.66	PGD	-2.61
SELENBP1	-1.71	LTBP1	-1.59	PKM	-1.82
SLC3A2	-2.56	MANF	-1.56	PPIB	-2.91
SPINK3	-1.84	MAP4	-1.88	PRDX5	-1.64

SRC	-2.13	MAPRE1	-1.52	PROCR	-2.40
TAGLN2	-1.70	MAPRE2	-1.55	RAB5A	-1.75
TES	-1.84	MTHFD1	-2.10	RALB	-1.56
TMOD2	-1.79	MUG2	-1.84	RNASE4	-2.51
TPI1	-1.86	MYH9	-1.69	SERPINA1	-1.75
TPM3	-1.56	NIT1	-2.43	SERPINA3K	-1.82
TPM4	-2.14	NUDC	-1.69	SERPINA3L	-1.64
UBB	-2.27	OAF	-1.71	SIAE	-1.95
VCL	-1.73	OLFM1	-2.07	SOD1	-2.39
VIM	-2.05	OSMR	-2.35	SPTAN1	-2.33
YWHAH	-1.79	PACSIN2	-1.85	STIP1	-2.00
		PAK2	-1.54	TGM1	-1.53
		PAM	-1.66	TPM2	-2.14
		PECAM1	-2.11	TTR	-1.70
		PFKL	-1.90	TUBB4B	-2.34
		PLEC	-1.72	TXNL1	-2.91
		PLG	-1.56	VAPA	-1.51
		PLOD1	-1.82	WFDC1	-1.79
		PODXL	-1.64	XDH	-1.52
		PPIF	-1.87	XK	-1.74
		PPP1R7	-4.43	YWHAB	-4.00
		PSMA6	-1.51	YWHAE	-2.40
		PSMB2	-1.52	YWHAG	-2.15

***S Table 8. The top 20 most differentially expressed transcripts in thyroid tissue**. Two transcripts were present in both young and adult rats (Dmkn and Krt13).*

Thyroid transcript				
	Young rats		Adult rats	
**Transcript**	**Fold Change**	**Group**	**Fold Change**	**Group**
Aqp3	-6,75	Y3		
Asprv1	-7,68	Y3		
Calml5	-8,32	Y3		
Csta	-7,04	Y3		
Defb4	-6,80	Y3		
Klk8	-7,97	Y3		
Krt13	-7,83	Y3	3,67	A6
Krt78	-7,31	Y3		
LOC102552128	-6,63	Y3		
Ly6d	-7,30	Y3		
Mt4	-8,38	Y3		
RGD1310935	-6,63	Y3		
RGD1560559	-7,58	Y3		
RGD1562234	-6,95	Y3		
Serpinb3a	-6,97	Y3		
Serpinb12	-7,60	Y3		
Slurp1	-6,44	Y3		
Stfa3	-7,71	Y3		
RGD1562885	6,59	Y6		
Dmkn	6,66	Y6	4,48	A3
Col17a1			3,80	A3
Ggcx			-4,66	A3
Hao2			-4,21	A3
Kap			-5,15	A3
Klk1c9			-4,03	A3
Lipf			-3,94	A3
Umod			-3,61	A3
Miox			-5,30	A3
Slpi			3,63	A3
Sst			5,62	A3
Aldh1a7			4,10	A9
Myh6			5,86	A9
Myh7			5,50	A9
Myl2			5,22	A9
Pth			-3,54	A9
Sln			-4,14	A9
Tnnt1			3,98	A9
Vegfb			-4,82	A9

***S Table 9. The top 20 most differentially expressed proteins in thyroid tissue**. Totally five proteins were found in both young and adult rats (*LYPD3, KRT4, KRT13, KRT14 and OCM*).*

Thyroid proteins				
	Young rats		Adult rats	
**Protein**	**Fold Change**	**Test group**	**Fold Change**	**Test group**
KRT13	-64,21	Y3	11,37	A3
KRT14	-3,93	Y3	5,21	A3
LYPD3	-4,81	Y3	4,95	A3
ADA	-13,48	Y3		
ALDH3A1	-4,50	Y3		
ANXA8	-3,98	Y3		
HMMR	-10,39	Y3		
KRT15	-4,61	Y3		
LGALS7	-11,00	Y3		
KRT4	4,50	Y6	6,95	A6
OCM	4,48	Y6	7,11	A3
A1M	-4,11	Y6		
COX6A2	-4,83	Y6		
DAP	-5,20	Y6		
GRPCB	4,58	Y6		
PSBPC2	15,18	Y6		
PTH	-6,24	Y6		
FASN	4,30	Y9		
Ig kappa chain C region, B allele	14,22	Y9		
RPN2	4,03	Y9		
ACARDS	-4,78	A3		
ADH5			-4,83	
GGT1			-5,42	A3
GSTA1			-7,18	A3
HADHB			-5,74	A3
IL17F			5,38	A6
Major urinary protein			-6,10	A3
ARCN1			4,92	A6
C3			7,15	A6
CACNG6			7,76	A6
COMP			4,78	A6
MAPK3			7,10	A6
RALA			7,17	A6
TFAM			-5,46	A6
COQ9			-11,21	A9

S Table 10. The top 20 most differentially expressed proteins in plasma. *Three proteins were found in both young and adult ratps* (A2M, RAD23B and RGS18).

Plasma proteins				
	Young rats		Adult rats	
**Protein**	**Fold Change**	**Test group**	**Fold Change**	**Test group**
RGS18	-9,02	Y3	-8,12	A9
DDT	-4,92	Y3		
FAH	-5,08	Y3		
GSTA2	-4,05	Y3		
RGN	-4,62	Y3		
RT1-AW2	4,55	Y3		
SPRR1A	4,01	Y3		
XK	9,68	Y3		
A2M	-17,63	Y6	-23,18	A3
RAD23B	-6,27	Y6	-6,59	A3
APOA2	-5,00	Y6		
C4BPB	-4,41	Y6		
CKM	-9,74	Y6		
FN1	-4,87	Y6		
HINT1	-4,14	Y6		
HP	-4,03	Y6		
PSBPC2	17,23	Y6		
PVALB	-5,65	Y6		
RNASE4	-5,59	Y6		
LGALS5	4,31	Y9		
DPEP2			-4,95	A3
MCPT1			-4,75	A6
ANXA1			7,39	A9
eEF1A1			8,10	A9
HIST1H1E			12,82	A9
HIST1H2BA			16,16	A9
Histone H2A type 3			11,01	A9
Histone H3.1			14,84	A9
HNRNPA2B1			9,35	A9
HNRNPC			17,58	A9
HNRNPK			5,74	A9
LMNA			10,95	A9
NRIF1			14,50	A9
PGAM1			4,88	A9
PRDX4			7,67	A9
SKAP2			-9,29	A9
VCP			6,84	A9

S Table 11. Significantly regulated pathways form ingenuity canonical pathways (IPA) of young and adult rats using the IPA software. *Activated (z>2.0) or inhibited (z>-2.0) signalling pathways, are presented.*

Ingenuity Canonical Pathway	p	z	Target molecules in dataset
**Y3**			
**Transcripts in thyroid**			
IL-6 Signalling	$1.9 \cdot 10^{-3}$	-2.0	IL1RN,KL,TNFRSF11B,IL36B
**Proteins in thyroid**			
Signalling by Rho Family GTPases	$3.4 \cdot 10^{-4}$	3.0	ACTR2,ACTR3,ARPC1B,PTPN11,MAPK3,SEPT7,ACTC1,MYL3,SEPT2
Integrin Signalling	$5.4 \cdot 10^{-7}$	-2.9	ACTR2,ACTR3,AKT1,ARPC1B,PTPN11,ARF3,MAPK3,RALB,ITGB4,GSN,ACTC1,RAP1A
Acute Phase Response Signalling	$2.5 \cdot 10^{-8}$	-2.6	C4A/C4B,PLG,ITIH3,HP,AKT1,TF,PTPN11,APCS,MAPK3,FGB,F2,SERPIND1
RhoA Signaling	$9.3 \cdot 10^{-5}$	-2.6	ACTR2,ACTR3,ARPC1B,SEPT7,ACTC1,MYL3,SEPT2
Ephrin Receptor Signalling	$1.0 \cdot 10^{-3}$	-2.6	ACTR2,ACTR3,AKT1,ARPC1B,PTPN11,MAPK3,RAP1A
ILK Signalling	$1.4 \cdot 10^{-3}$	-2.6	AKT1,PTPN11,MAPK3,KRT18,ITGB4,ACTC1,MYL3
FcÎ³ Receptor-mediated Phagocytosis in Macrophages and Monocytes	$1.9 \cdot 10^{-4}$	-2.5	ACTR2,ACTR3,AKT1,ARPC1B,MAPK3,ACTC1
Thrombin Signalling	$9.6 \cdot 10^{-3}$	-2.5	AKT1,CAMK1,PTPN11,MAPK3,MYL3,F2
Actin Cytoskeleton Signalling	$1.8 \cdot 10^{-4}$	-2.3	ACTR2,ACTR3,ARPC1B,PTPN11,MAPK3,GSN,ACTC1,MYL3,F2
Remodeling of Epithelial Adherens Junctions	$1.6 \cdot 10^{-10}$	-2.2	ACTR2,TUBB3,RAB5A,ACTR3,ARPC1B,TUBB2A,RAB7A,TUBA1C,TUBB,ACTC1
Regulation of Actin-based Motility by Rho	$1.2 \cdot 10^{-4}$	-2.2	ACTR2,ACTR3,ARPC1B,GSN,ACTC1,MYL3
Rac Signalling	$4.2 \cdot 10^{-4}$	-2.2	ACTR2,ACTR3,ARPC1B,PTPN11,MAPK3
fMLP Signalling in Neutrophils	$4.7 \cdot 10^{-4}$	-2.2	ACTR2,ACTR3,ARPC1B,PTPN11,MAPK3
GÎ±12/13 Signalling	$6.2 \cdot 10^{-4}$	-2.2	AKT1,PTPN11,MAPK3,MYL3,F2
Insulin Receptor Signalling	$8.1 \cdot 10^{-3}$	-2.2	AKT1,PTPN11,MAPK3,PPP1CA,EIF2B2
**Proteins in plasma**			
RhoA Signalling	$6.0 \cdot 10^{-4}$	2.2	PFN1,CFL1,ARPC2,EZR, MYL12B
**Y6**			
**Proteins in thyroid**			
LXR/RXR Activation	$3.6 \cdot 10^{-4}$	-2.8	C4A/C4B,PON1,C3,APOA1,APOA2,CD36,AMBP,HADH,APOD
EIF2 Signalling	$2.7 \cdot 10^{-9}$	-2.6	RPL24,EIF3H,RPL3,RPS8,RPL17,EIF4A2,EIF3E,EIF2S1,RPL9,RPL10A,RPS7,RPS4Y1,RPS16,PTPN11,EIF3B,RPS27L,RPL10,RPS2,RPS3
PPARÎ±/RXRÎ± Activation	$7.9 \cdot 10^{-3}$	-2.4	ACADL,APOA1,GPD2,APOA2,CD36,PRKACA,GNAQ,GOT2,AP2A2
Ephrin Receptor Signalling	$2.3 \cdot 10^{-2}$	-2.2	GNAI2,GNB1,PTPN11,ARPC2,RACK1,GNB2,GNAQ,RAP1A
Acute Phase Response Signalling	$2.4 \cdot 10^{-7}$	-2.1	ITIH3,C3,APOA2,AMBP,CP,FGG,SERPIND1,C4A/C4B,KLKB1,HMOX1,HP,APOA1,PTPN11,APCS,FGB
Actin Cytoskeleton Signalling	$4.6 \cdot 10^{-3}$	-2.1	MYL9,MYH4,MYH6,MYH9,PTPN11,MYH8,ARPC2,MYH3,MYH7,MYH11,MYL3
**Proteins in plasma**			
LXR/RXR Activation	$3.2 \cdot 10^{-7}$	-2.5	APOE,TTR,APOA1,APOM,APOH,APOC4,APOA2,PCYOX1,LBP,APOD

Production of Nitric Oxide and Reactive Oxygen Species in Macrophages	$1.6\cdot10^{-4}$	-2.3	APOE,APOA1,APOM,PTPN11,APOC4,APOA2,CAT,PCYOX1,APOD
Regulation of Actin-based Motility by Rho	$3.0\cdot10^{-4}$	2.2	ACTR3,PFN1,CFL1,ACTA2,ACTB,ARHGDIA
RhoA Signalling	$8.5\cdot10^{-3}$	2.2	ACTR3,PFN1,CFL1,ACTA2,ACTB
FcÎ³ Receptor-mediated Phagocytosis in Macrophages and Monocytes	$4.8\cdot10^{-4}$	2.4	SRC,ACTR3,ACTA2,ACTB,RAB11A,CRK
Colorectal Cancer Metastasis Signalling	$4.8\cdot10^{-2}$	2.4	SRC,CDH1,PTPN11,TGFB1,PRKACA,MMP2
Paxillin Signalling	$2.0\cdot10^{-4}$	2.6	SRC,PTPN11,ACTA2,ACTB,CRK,VCL,ACTN1
Leukocyte Extravasation Signalling	$3.5\cdot10^{-4}$	2.8	SRC,TIMP3,PTPN11,ACTA2,ACTB,CRK,MMP2,VCL,ACTN1
Integrin Signalling	$3.2\cdot10^{-6}$	3.3	SRC,ACTR3,PFN1,PTPN11,ACTA2,ACTB,CAPN1,RALB,CRK,CAPN2,VCL,ACTN1

Y9

Proteins in thyroid

PTEN Signalling	$1.6\cdot10^{-3}$	-2.6	IKBKB,CSNK2A1,MAPK1,MAPK3,RAC1,KRAS,DDR1
Integrin Signalling	$7.4\cdot10^{-5}$	2.1	PARVA,PFN1,MAPK1,ARF3,ARPC2,MAPK3,ACTB,Wasl,RAC1,CRK,KRAS,RAP1A
Leukocyte Extravasation Signalling	$1.2\cdot10^{-2}$	2.1	GNAI2,MAPK1,EZR,ACTB,Wasl,RAC1,CRK,RAP1A
Calcium Signalling	$8.5\cdot10^{-4}$	2.2	TNNI2,MAPK1,MAPK3,CASQ1,TNNI1,RAP1A,Tpm2,CASQ2,ATP2A1
STAT3 Pathway	$3.4\cdot10^{-3}$	2.2	MAPK1,MAPK3,RAC1,KRAS,DDR1
NRF2-mediated Oxidative Stress Response	$6.8\cdot10^{-3}$	2.2	AKR1A1,SOD2,DNAJC5,MAPK1,MAPK3,ACTB,VCP,KRAS
Î±-Adrenergic Signalling	$8.5\cdot10^{-3}$	2.2	GNAI2,PYGM,MAPK1,MAPK3,KRAS
LPS-stimulated MAPK Signalling	$8.9\cdot10^{-3}$	2.2	IKBKB,MAPK1,MAPK3,RAC1,KRAS
PDGF Signalling	$1.1\cdot10^{-2}$	2.2	CSNK2A1,MAPK1,MAPK3,CRK,KRAS
HGF Signalling	$2.8\cdot10^{-2}$	2.2	MAPK1,MAPK3,RAC1,KRAS,RAP1A
Sphingosine-1-phosphate Signalling	$2.9\cdot10^{-2}$	2.2	GNAI2,MAPK1,MAPK3,CASQ1,RAC1
Rac Signalling	$2.8\cdot10^{-2}$	2.2	MAPK1,ARPC2,MAPK3,RAC1,KRAS
GNRH Signalling	$4.2\cdot10^{-2}$	2.2	GNAI2,MAPK1,MAPK3,RAC1,KRAS
GÎ±12/13 Signalling	$4.2\cdot10^{-2}$	2.2	IKBKB,CDH2,MAPK1,MAPK3,KRAS
Ephrin Receptor Signalling	$1.1\cdot10^{-3}$	2.3	GNAI2,MAPK1,ARPC2,MAPK3,Wasl,RAC1,CRK,KRAS,RAP1A
Renal Cell Carcinoma Signalling	$1.5\cdot10^{-4}$	2.4	MAPK1,MAPK3,RAC1,CRK,KRAS,FH,RAP1A
Paxillin Signalling	$6.2\cdot10^{-3}$	2.4	PARVA,MAPK1,ACTB,RAC1,CRK,KRAS
fMLP Signalling in Neutrophils	$8.5\cdot10^{-3}$	2.4	GNAI2,MAPK1,ARPC2,MAPK3,RAC1,KRAS
PKCÎ¸ Signalling in T Lymphocytes	$1.0\cdot10^{-2}$	2.4	IKBKB,MAPK1,MAPK3,RAC1,KRAS,HLA-DQB1
p70S6K Signalling	$1.2\cdot10^{-2}$	2.4	GNAI2,MAPK1,EEF2,MAPK3,KRAS,EEF2K
CXCR4 Signalling	$3.4\cdot10^{-2}$	2.4	GNAI2,MAPK1,MAPK3,RAC1,CRK,KRAS
Role of NFAT in Regulation of the Immune Response	$4.2\cdot10^{-2}$	2.4	GNAI2,IKBKB,MAPK1,MAPK3,KRAS,HLA-DQB1

B Cell Receptor Signalling	$4.7\cdot10^{-2}$	2.4	IKBKB,MAPK1,MAPK3,RAC1,KRAS,RAP1A
Signalling by Rho Family GTPases	$8.7\cdot10^{-4}$	2.5	GNAI2,SEPT8,CDH2,MAPK1,ARPC2,EZR,MAPK3,ACTB,SEPT7,RAC1,SEPT2
RhoA Signalling	$1.7\cdot10^{-3}$	2.6	SEPT8,PFN1,ARPC2,EZR,ACTB,SEPT7,SEPT2
NGF Signalling	$1.7\cdot10^{-3}$	2.6	IKBKB,MAPK1,MAPK3,RAC1,CRK,KRAS,RAP1A
FcÎ³ Receptor-mediated Phagocytosis in Macrophages and Monocytes	$6.5\cdot10^{-5}$	2.8	MAPK1,RAB11B,ARPC2,EZR,MAPK3,ACTB,RAC1,CRK
Actin Cytoskeleton Signalling	$1.5\cdot10^{-3}$	3.0	KNG1,PFN1,MAPK1,ARPC2,EZR,MAPK3,ACTB,RAC1,CRK,KRAS

A3

Proteins in thyroid

EIF2 Signalling	2.5·10-11	-3.2	RPL11,RPS18,RPL35A,EIF4A2,RPS7,RPL14,RPL13,EIF3B,RPL18A,RPS2,RPS3, RPL18,PABPC1,EIF3H,RPL3,RRAS,RPS8,RPL17,EIF3J,RPL12, EIF3E,RPL10A,RPL9,RPS4Y1,PTPN11,RPL10,RPS14
Acute Phase Response Signalling	6.9·10-5	-3.0	ITIH3,FN1,C3,RRAS,APOA2,CP,SERPIND1,PLG,C4A/C4B,HMOX1,HP, APOA1,PTPN11,TF,APCS,FGB
Ephrin Receptor Signalling	1.1·10-2	-2.5	GNAI2,ACTR2,GNAS,ARPC1B,PTPN11,RRAS,SDC2,ARPC2,RACK1,ADAM10,RAC1,RAP1A
LXR/RXR Activation	3.2·10-3	-2.3	C4A/C4B,PON1,C3,APOA1,TF,APOA2,CD36,S100A8,HADH,CLU
fMLP Signalling in Neutrophils	5.6·10-3	-2.3	GNAI2,ACTR2,GNAS,ARPC1B,PTPN11,RRAS,ARPC2,PPP3R1,RACK1, RAC1
ERK/MAPK Signalling	9.3·10-3	-2.3	RRAS,HSPB2,RAC1,PPP1R11,RAP1A,PRKAG1,EIF4EBP1,YWHAQ, H3F3A/H3F3B,PTPN11,PRKACA,PRKAR1A,HSPB1
Integrin Signalling	5.9·10-4	-2.2	RAP2B,ACTR2,ARPC1B,MYL2,RRAS,RALB,RAC1,GSN,RAP1A,MYL9, ARF5,PTPN11,ARF3,ARPC2,CAPN1,CAV1,VCL
Calcium Signalling	1.2·10-4	-2.1	RAP2B,MYH4,MYL2,Tpm1,Tpm2,RAP1A,MYL1,PRKAG1,ATP2A1,MYL9, PPP3R1,CASQ1,PRKACA,CASQ2,MYL3,PRKAR1A
PPARÎ±/RXRÎ± Activation	4.5·10-3	-2.1	ACADL,GNAS,APOA1,HSP90AB1,RRAS,GPD2,APOA2,CD36,PRKACA, GOT2,AP2A2,PRKAG1,PRKAR1A
CDK5 Signalling	1.2·10-2	-2.1	GNAS,PPP1R1B,RRAS,CAPN1,PRKACA,PPP1R11,PRKAG1,PRKAR1A
Cardiac Î²-adrenergic Signalling	2.6·10-2	-2.1	GNAS,PPP1R1A,RACK1,PRKACA,PPP1R11,PKIA,PRKAG1,PRKAR1A, ATP2A1
Phospholipase C Signalling	4.9·10-4	2.2	GNAS,PLD3,MYL2,RRAS,MYLPF,RALB,RACK1,RAC1,RAP1A,MYL1, MYL9,HMOX1,PPP3R1,FCER1G,MYL3

Proteins in plasma

FcÎ³ Receptor-mediated Phagocytosis in Macrophages and Monocytes	2.5·10-6	2.1	SRC,ACTR3,ACTA2,ARPC2,EZR,ACTB,RAB11A,CRK
RhoA Signalling	1.5·10-5	2.1	ACTR3,PFN1,CFL1,ACTA2,ARPC2,EZR,ACTB,MYL12B
Leukocyte Extravasation Signalling	1.3·10-4	2.1	SRC,PTPN11,ACTA2,PLCG2,EZR,ACTB,CRK,VCL,ACTN1
Melanocyte Development and Pigmentation Signalling	2.0·10-3	2.2	SRC,PTPN11,PLCG2,PRKACA,CRK
Neuropathic Pain Signalling In Dorsal Horn Neurons	4.3·10-3	2.2	SRC,PTPN11,PDIA3,PLCG2,PRKACA
PI3K/AKT Signalling	5.5·10-3	2.2	YWHAQ,HSP90B1,YWHAH,YWHAZ,ILK
Ephrin Receptor Signalling	6.0·10-3	2.2	SRC,ACTR3,PTPN11,CFL1,ARPC2,CRK
Cdc42 Signalling	7.6·10-3	2.2	SRC,ACTR3,CFL1,ARPC2,MYL12B

Tec Kinase Signalling	1.9·10-2	2.2	SRC,PTPN11,ACTA2,PLCG2,ACTB
Role of NFAT in Cardiac Hypertrophy	3.4·10-2	2.2	SRC,PTPN11,PDIA3,PLCG2,PRKACA
Thrombin Signalling	4.0·10-2	2.2	SRC,PTPN11,PDIA3,PLCG2,MYL12B
Regulation of Actin-based Motility by Rho	1.4·10-5	2.4	ACTR3,PFN1,CFL1,ACTA2,ARPC2,ACTB,MYL12B
Paxillin Signalling	2.9·10-5	2.6	SRC,PTPN11,ACTA2,ACTB,CRK,VCL,ACTN1
Integrin Signalling	1.3·10-8	3.6	SRC,PFN1,ACTB,ILK,CRK,ACTR3,ACTA2,PTPN11,ARPC2,PLCG2, CAPN2,VCL,MYL12B,ACTN1

A6

Proteins in thyroid

Agrin Interactions at Neuromuscular Junction	4.9·10-3	2.2	MAPK3,ACTB,ACTC1,CTTN,ACTA1
Paxillin Signalling	3.5·10-2	2.2	ACTB,ACTN4,ACTC1,ACTA1,ACTN1
VEGF Signalling	6.3·10-3	2.4	MAPK3,ACTB,ACTN4,ACTC1,ACTA1,ACTN1
Leukocyte Extravasation Signalling	1.8·10-2	2.6	CRKL,ACTB,ACTN4,ACTC1,RAP1A,CTTN,ACTA1,ACTN1
ILK Signaling	3.1·10-2	2.6	MYH9,MAPK3,ACTB,ACTN4,ACTC1,ACTA1,ACTN1
Integrin Signalling	1.9·10-4	3.3	PFN1,RALA,CRKL,MAPK3,ACTB,ACTN4,GSN,ACTC1,RAP1A,CTTN, ACTA1,ACTN1

A9

Transcripts in thyroid

Dopamine Receptor Signalling	1.5·10-2	-2.0	PRKACB,PPP2R3A,PPP1CB,PPP1R3A,PTH

Proteins in thyroid

Actin Cytoskeleton Signalling	7.9·10-4	2.2	MYL2,MYLK2,MYH7,VCL,MYL3,ACTN1

Proteins in plasma

HIPPO signalling	1.3·10-3	-2.2	YWHAQ,YWHAG,YWHAE,YWHAB,YWHAZ
14-3-3-mediated Signalling	4.5·10-6	2.2	YWHAQ,YWHAG,YWHAE,TUBB4B,YWHAB,TUBB2A,YWHAZ,TUBA4A, TUBA1C
PI3K/AKT Signalling	2.6·10-5	2.8	YWHAQ,HSP90B1,YWHAG,YWHAE,HSP90AB1,YWHAB,YWHAZ, HSP90AA1
